# Upregulation of *BCL-2* by acridone derivative through gene promoter i-motif for alleviating liver damage of NAFLD/NASH

**DOI:** 10.1093/nar/gkaa615

**Published:** 2020-07-25

**Authors:** Xiaoya Li, Jing Wang, Xue Gong, Meiling Zhang, Shuangshuang Kang, Bing Shu, Zuzhuang Wei, Zhi-Shu Huang, Ding Li

**Affiliations:** School of Pharmaceutical Sciences, Sun Yat-sen University, Guangzhou University City, 132 Wai huan East Road, Guangzhou 510006, P. R. China; School of Pharmaceutical Sciences, Sun Yat-sen University, Guangzhou University City, 132 Wai huan East Road, Guangzhou 510006, P. R. China; School of Pharmaceutical Sciences, Sun Yat-sen University, Guangzhou University City, 132 Wai huan East Road, Guangzhou 510006, P. R. China; School of Pharmaceutical Sciences, Sun Yat-sen University, Guangzhou University City, 132 Wai huan East Road, Guangzhou 510006, P. R. China; School of Pharmaceutical Sciences, Sun Yat-sen University, Guangzhou University City, 132 Wai huan East Road, Guangzhou 510006, P. R. China; School of Pharmaceutical Sciences, Sun Yat-sen University, Guangzhou University City, 132 Wai huan East Road, Guangzhou 510006, P. R. China; School of Pharmaceutical Sciences, Sun Yat-sen University, Guangzhou University City, 132 Wai huan East Road, Guangzhou 510006, P. R. China; School of Pharmaceutical Sciences, Sun Yat-sen University, Guangzhou University City, 132 Wai huan East Road, Guangzhou 510006, P. R. China; School of Pharmaceutical Sciences, Sun Yat-sen University, Guangzhou University City, 132 Wai huan East Road, Guangzhou 510006, P. R. China

## Abstract

Nonalcoholic fatty liver disease (NAFLD)/nonalcoholic steatohepatitis (NASH) are global epidemic public health problems with pathogenesis incompletely understood. Hepatocyte excessive apoptosis is a significant symbol for NAFLD/NASH patients, and therefore anti-apoptosis therapy could be used for NAFLD/NASH treatment. Up-regulation of *BCL-2* has been found to be closely related with anti-apoptosis. *BCL-2* gene promoter region has a C-rich sequence, which can form i-motif structure and play important role in regulating gene transcription. In this study, after extensive screening and evaluation, we found that acridone derivative **A22** could up-regulate *BCL-2* transcription and translation *in vitro* and in cells through selective binding to and stabilizing *BCL-2* gene promoter i-motif. Our further experiments showed that **A22** could reduce hepatocyte apoptosis in NAFLD/NASH model possibly through up-regulating *BCL-2* expression. **A22** could reduce inflammation, endoplasmic reticulum stress and cirrhosis in high-fat diet-fed mice liver model. Our findings provide a potentially new approach of anti-apoptosis for NAFLD/NASH treatment, and **A22** could be further developed as a lead compound for NAFLD/NASH therapy. Our present study first demonstrated that gene promoter i-motif could be targeted for gene up-regulation for extended treatment of other important diseases besides cancer.

## INTRODUCTION

Nonalcoholic fatty liver disease (NAFLD) is a main course of liver disease including a wide spectrum of disorders ranged from steatosis to steatohepatitis. Around 30% of patients with NAFLD have nonalcoholic steatohepatitis (NASH) (1), which is featured by liver injury, inflammation and fibrosis (2,3). NAFLD is also an important component of metabolic syndrome, which has enormous risk of running into cirrhosis or hepatocellular carcinoma. However, specific pathological mechanism of NASH is incompletely understood (4), and there is no specific pharmacological targeted drugs for NASH treatment (5,6). Lifestyle interventions with diet and exercise to achieve weight loss goal remain the cornerstone of treatment for NAFLD and NASH (7). Thus, effective targeted therapy is urgently required.

Hepatocyte apoptosis has been proved to be a generally prominent feature of NAFLD patients (8). *BCL-2* family modulates the checkpoint of apoptosis and has two major category members: apoptosis members like Bax, and anti-apoptosis members like *BCL-2* (9). The anti-apoptotic *BCL-2* expression has been proved to be diminished in both hepatocytes and serum in accordance with stage of NAFLD/NASH (10–12). Therefore, we hypothesized that up-regulation of *BCL-2* expression could play important anti-hepatocyte apoptosis role in the course of NAFLD. It has been shown that stabilization of *BCL-2* gene promoter i-motif could up-regulate gene transcription and translation (13,14), and other gene promoter i-motifs have also been shown to play crucial roles in regulating gene transcription and translation (15). i-Motif structure is formed by C–C base pairs (C+:C) stacking force (16), and is stabilized in faintly acidic (17) or molecular crowding condition *in vitro* (18). It has been shown that i-motif can form in telomeres (19–22), promoter regions of proto-oncogenes (23), centromeres (24,25) by using biophysical techniques. Recently, it has been further demonstrated that i-motif structures can form in promoters and telomeric regions by using i-motif specific antibodies (19). We have also revealed that an acridone derivative could selectively bind to oncogene *C-MYC* promoter i-motif and down-regulate gene transcription and translation (26).

In this study, after extensive screening of our small molecule library, an acridone derivative **A22** was found to interact with the C-rich sequence in P1 promoter of *BCL-2*, which significantly up-regulated *BCL-2* gene transcription and translation. **A22** could bind to and stabilize the i-motif structure formed by the C-rich sequence, and then possibly recruit hnRNP LL protein to form a complex promoting transcription and translation. Besides, we evaluated therapeutic effect of **A22** on the pathological apoptotic characteristics of NASH in mice model of NAFLD/NASH generated by prolonged feeding with a high-fat diet. The i-motif binding ligand **A22** showed significant therapeutic effect for NAFLD model by reducing hepatocyte apoptosis, affecting expressions of caspase family members (cleaved-caspase 3&9), hepatic injury (hepatocyte ballooning, serous levels of ALT and AST), hepatic inflammatory response (inflammatory cytokines and lymphocyte mononuclear macrophage numbers) possibly through up-regulating *BCL-2*.

As we know, this is the first effort to target gene promoter i-motif with a small molecule for NAFLD/NASH treatment. It should be mentioned that gene promoter G-rich sequence could form G-quadruplex, which has been widely studied as potential target for cancer treatment, possibly because its stabilization by small molecule normally down-regulate gene transcription. In comparison, stabilization of gene promoter i-motifs by small molecules have been found to either down-regulate or up-regulate gene transcriptions, possibly due to their different binding affinity to transcription factors. Therefore, up-regulation of certain gene transcription through its promoter i-motif could possibly be developed for treatment of other important diseases with reasonably good efficiency and selectivity.

## MATERIALS AND METHODS

### General materials

Chemically synthesized DNA oligomers of HPLC purified grade were purchased from Sangon Biotec, as shown in Supplementary Table S1. All oligonucleotide concentrations were determined by measuring absorbance at 260 nm using a Nano Drop1000 spectrophotometer (Thermo Scientific). For obtaining i-motif structures, C-rich oligonucleotides were annealed in 1× BPES buffer (30 mM KH_2_PO_4_, 30 mM K_2_HPO_4_, 1 mM EDTA, 100 mM KCl) with different pH at 95°C for 5 min, and then cooled to room temperature. For obtaining G-quadruplex, oligonucleotides were annealed in 20 mM Tris–HCl buffer containing 100 mM KCl (pH 7.4) by heating at 95°C for 5 min followed with gradual cooling to room temperature. Formation of secondary non-B DNA structures were determined by using circular dichroism (CD) spectrophotometer. *BCL-2* and other oncogene promoter i-motif structures had a positive peak at 287 nm and a negative peak around 260 nm. *BCL-2* promoter G-quadruplex had a positive peak at 263 nm and a negative peak around 240 nm. Cells were purchased from American Type Culture Collection (ATCC, China). All cell culture reagents including DCFH-DA (D-6883), BSA-conjugated palmitic acid (PA, CID: 985), and BSA-conjugated oleic acid (OA, CID: 445639), were purchased from Sigma–Aldrich. Antibodies were purchased from Affinity Company. Annexin V-FITC/PI apoptosis kit (70-AP101-100) was purchased from Muti-sciences. High-fat diet (HF, 60% of calories from fat) was purchased from Research Diet (Catalog number D12492, New Brunswick, NJ, USA) with detailed information listed in supplementary information. Synthetic route for acridone derivative **A22** and **A22-HCl** was shown in Supplementary Scheme I.

### Surface plasmon resonance (SPR)

Surface plasmon resonance (SPR) measurements were conducted by using a GLH sensor chip on a ProteOn XPR-36 Protein Interaction Array system (Bio-Rad Laboratories, Hercules, CA, USA). 5′-Biotin labeled DNAs were annealed in different buffers, and then immobilized (∼12 000 RU) in flow cells. In screening experiment, all ligands were diluted to different concentrations with running buffers (i-motif running buffer: 20 mM MES, 100 mM KCl, pH 5.5; G-quadruplex running buffer: 20 mM Tris–HCl, 100 mM KCl, pH 7.4). Ligands of different concentrations were injected at flow rate of 25 μl/min for 240 s of association phase, and 300 s of disassociation phase at 25°C. The GLH sensor chip was regenerated with short injection of 50 mM NaOH solution between consecutive measurements. The final graphs were obtained by subtracting blank sensorgram of corresponding i-motif or G-quadruplex. Kinetic and equilibrium measurements were analyzed with ProteOn manager software. Langmuir model was used to fit SPR kinetic sensorgrams. *K*_D_ values were obtained through equation *K*_D_ = *k*_d_*/k*_a_. For association process, *d*[AL]/*d_t_* = *k*_a_[A][L] – *k*_d_[AL], and for disassociation process [A] = 0, *d*[AL]/*d_t_* = -*k*_d_[AL] (*t* means time, [A] is concentration of analyte, [L] is concentration of substance and [AL] is concentration of complex).

### Nuclear magnetic resonance

Nuclear magnetic resonance (^1^H NMR) experiments were carried out at 298 K by using a Bruker Avance 600 NMR spectrometer equipped with a 5 mm TCI Micro CyroProbe. The 3–9–19 WATERGATE gradient spin echo was used to suppress water solvent signal. The number of scans in the experiments was 256 by double scan. All spectra were collected using a spectral width of 18 000 Hz (30 ppm), with an alternate acquisition time of 4 s and a relaxation delay of 6 s. The DNA samples were annealed to 500 μM concentration with or without different equivalent of **A22** in 1× BPES buffer of different pH before experiments.

### Circular dichroic (CD) spectroscopy and CD-melting experiments

CD measurements were performed on a Chirascan circular dichroism spectrophotometer (Chirascan, England). A 10 mm path length quartz cuvette was used to record the spectra with a wavelength range of 230–350 nm with a 1 nm bandwidth. The oligonucleotides had wild type unmodified DNA sequences, with py39 for *BCL2* promoter i-motif, and pu39 for *BCL2* promoter G-quadruplex. Other oligonucleotides for oncogene promoter i-motifs were simplified with their corresponding names, such as *C-MYC*, *C-KIT, KRAS* and *VEGF*. CD and CD melting experiments were performed at a fixed i-motif or G-quadruplex concentration (1 μM) either with or without a fixed concentration (20 μM) of **A22** in different buffers. Data collection was performed at intervals of 5°C over a range of 25–95°C with a heating rate of 1°C/min.

### Fluorescence titrations

Fluorescence titrations were performed on the HORIBA Jobin Yvon FluoroMAX-4 spectrofluorometer. Py39 dual labeled with 5′-FAM and 3′-TAMRA of 1 μM concentration was successively titrated with different equivalent of **A22** in buffer of different pH. Then dose-dependent spectra changes at 516 nm for various dual labeled oligomers were recorded when increasing concentration of **A22**.

Fluorescent intercalator displacement (FID) assays using thiazole orange (TO) was a simple way to screen ligands against DNA secondary structures. For thiazole orange displacement, 1 μM annealed DNA was pre-incubated with 1 μM thiazole orange for 1 h at 37°C, and then different concentrations of **A22** were added into the solution. After each addition, the reaction was stirred and allowed to equilibrate for at least 5 min, and the fluorescence scan was taken with excitation wavelength at 500–700 nm.

### Cell culture and MTT

The following cell lines including cervix cancer cell line Siha, epithelial cancer cell line MCF7, lung adenocarcinoma cell line A549, osteosarcoma cell line U2OS, human hepatocellular carcinoma cell line HepG2, human pancreatic cancer cell PANC-1, human colon cancer cell line HCT116, human glioblastoma cell line U87MG, human bladder cancer cell line T24, human bladder transitional cancer cell line UM-UC-3, rat islet cell tumor cell line INS-1, mouse hippocampal neuron cell line HT22, human embryonic kidney cell line HEK293, human hepatic stellate cell line LX-2, were cultured in Dulbecco's modified Eagle's medium (DMEM) or RPMI 1640, with 10% fetal bovine serum at 37°C in 5% CO_2_. Cells were seeded in 96-well plates (5000 per well), and incubated with various concentrations of **A22** at 37°C in a humidified atmosphere of 5% CO_2_ for 24 h. Then 100 μl culture medium containing 2.5 mg/ml methyl thiazolyl tetrazolium (MTT) solution was added to each well for further incubation for 4 h. The cells in each well were treated with dimethyl sulfoxide (DMSO) (200 μl for each well) and the optical density (OD) was recorded at 570 nm. All drug doses were parallel tested in triplicate. The cytotoxicity was evaluated based on the percentage of cell survival in a dose-dependent manner comparing to the negative untreated control. Blank group wells just contained culture medium without cells. Cell viability was calculated with the following equation:

$$\begin{eqnarray*} {\rm{cell\, viability}}( \% ) = \frac{\left( {{\rm{OD\, of\, treated\, cells - OD\, of\, blank}}} \right)}{\left( {{\rm{OD\, of\, control\, cells - OD\, of\, blank}}} \right) \times 100} \end{eqnarray*}$$

HepG2 cells were cultured in DMEM with 10% fetal bovine serum and 10 U/ml penicillin/streptomycin (Thermo, Guangzhou, China) at 37°C in a humidified incubator with 5% CO_2_. Cells were seeded and overnight cultured. After the cell density attained to almost 70%, the cells were incubated with 0.5 mM oleic acid (OA, Sigma, China) or 0.5 mM palmitic acid (PA, Sigma, China) for 24 h for cell models, and then harvested for subsequent analysis. Oil red O staining and TG detection method were described in the Supplementary Methods section.

### Reverse transcription and real-time PCR

HepG2 cells were treated with or without **A22**, and corresponding total RNAs were isolated and purified. Reverse transcription was performed by using cDNA synthesis kit. The relative transcription levels for genes were measured with quantitative real-time PCR using SYBR Green-based method as described in the Supplementary Methods section. The primer sequences information and raw data were shown in Supplementary Tables S5 and S7.

### Protein extracts and western blotting

Cells after 24 h treatment with or without **A22** were harvested, and the proteins were extracted and quantified by using BCA Protein Quantitation Kit (Thermo Fish). Western blots were performed as described in the Supplementary Methods section.

### Animal experiment

Eight weeks aged male C57/B6J mice, with weight of 18–22 g, were bred at the Laboratory Animal Center of Sun Yat-sen university (Guangzhou, China) for experimental use. All procedures were approved by the Animal Care and Use Committee of Sun Yat-sen University and conformed to the legal mandates and national guidelines for the care and maintenance of laboratory animals. Animals were housed in specific pathogen free environment with stationary temperature at 22 ± 1°C on a 12 h dark/light cycle. After 1 week of adaptive feeding, the mice were divided into two groups: chow diet group fed with chow diet (CH, Animal Center proved 70% calories from starch), and high-fat diet group fed with high-fat diet (HF, 60% calories from fat purchased from Research Diet) for 16 weeks. CH and HF groups were randomly divided into subgroups at the beginning of week 10. The HF group had two subgroups treated with different dosage of **A22**: 10 mg/kg and 40 mg/kg. The CH group was treated with 40 mg/kg **A22** as a negative control. **A22** was dissolved in normal saline and injected through intraperitoneal injection (i.p.) every other day. The body weight and food intake were daily monitored.

### Serum biochemical test

The mice were fasted for 8 h, and then anesthetized with 10 mg/kg xylazine and 80 mg/kg ketamine through i.p. injection. After the mice were completely anesthetized, we withdrew blood, removed eyeball, and then had cervical dislocation. We weighted and took photos of target tissues and organs, and then freeze-clamped or fixed with 4% paraformaldehyde solution. Blood samples were centrifuged at the speed of 3000g for 15 min at 4°C, and then serums were collected for analysis (AST, AST, ALP, CHO, TG, Glucose, insulin, etc.).

### Histomorphological examination

Tissues fixed with 4% paraformaldehyde were dehydrated gradually with ethanol (70–100%), and then put on paraffin. The tissues were incised at 4 μm thick by a rotary microtome (Thermo, USA) for hematoxylin and eosin (H&E), masson and Sirius red staining. Steatosis, inflammation, ballooning, and fibrosis in liver were assessed with NAFLD activity scoring system. Sirius red staining was carried out to detect collagen, and masson staining was for fibrosis of liver. Monocyte-derived macrophages and lymphocyte were assessed by immunohistochemistry labeled with CD11b antibody and CD45 antibody, respectively. The apoptosis factors BCL-2 and cleaved-caspase 3 were evaluated by using immunohistochemistry with corresponding antibodies.

### Statistical analysis

Data were expressed as the mean ± SEM. Data between two groups were analyzed with unpaired Student's t-tests using Graphpad Prism (Graphpad Software Inc, California, USA). A *P value of ≤0.05 is considered statistically significant.

## RESULTS

### Screening of small molecules for binding to *BCL-2* gene promoter i-motif

**Figure 1.**
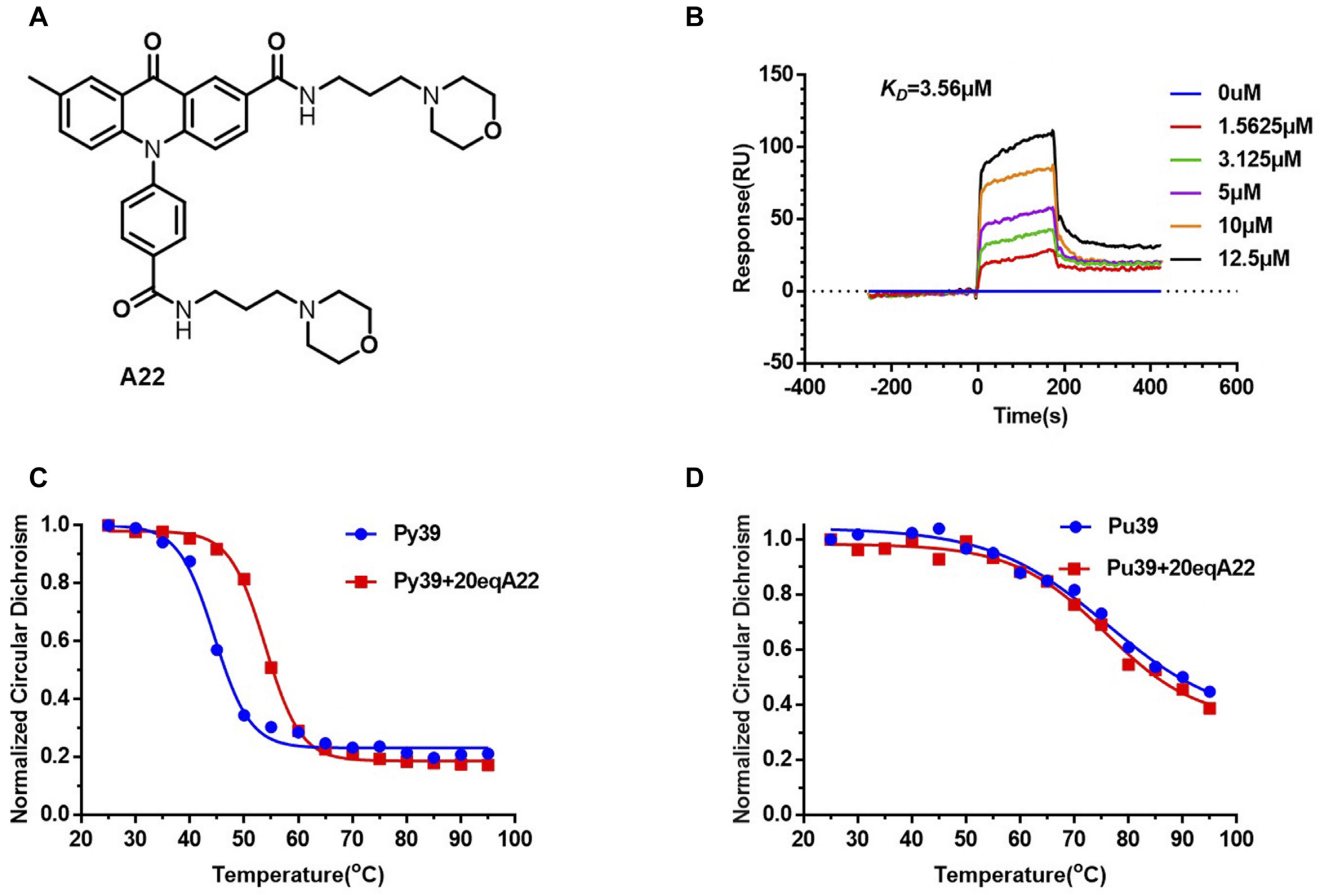
**A22** could specifically bind to and stabilize *BCL-2* gene promoter i-motif. (**A**) Structure of compound **A22**. (**B**) Binding affinity of **A22** to *BCL-2* gene promoter i-motif was studied by using SPR with *K*_D_ value determined to be 3.56 μM. Bio-py39 was annealed in MES buffer at pH 5.5 for this experiment. (**C**) CD melting experiment was recorded at 288 nm for py39 annealed in 1× BPES buffer at pH 5.5 with or without **A22**, indicating that **A22** could stabilize *BCL-2* gene promoter i-motif. Py39 had its maximum absorption at 288 nm. (**D**) CD melting experiment was recorded at 266 nm for pu39 with or without **A22**, indicating that **A22** had no significant effect on *BCL-2* promoter G-quadruplex. Pu39 had its maximum absorption at 266 nm, which was annealed in buffer of 20 mM Tris–HCl, 100 mM KCl, pH 7.4. All the experiments were repeated for three times.

In order to find a small molecule interacting with *BCL-2* gene promoter i-motif, we screened our small molecule library by using SPR. Molecules screened here include acridone derivatives, isaindigotone derivatives, benzo[*c*]acridine derivatives, bisacridine derivatives, and imidazole derivatives. The *K*_D_ values for interaction of the i-motif with various small molecules were determined as shown in Supplementary Table S2. Among these compounds, acridone derivative **A22** (Figure 1A) exhibited strong binding affinity to *BCL-2* gene promoter i-motif, with its *K*_D_ value determined to be 3.56 μM (Figure 1B). We then selected some of the outstanding compounds for Thiazole Orange (TO) fluorescent intercalator displacement assay (27) to further evaluate their binding affinity to *BCL-2* promoter i-motif formed with C-rich sequence py39. Our results (Supplementary Table S3, Supplementary Figure S2) showed that **A22** could significantly replace Thiazole Orange in binding with *BCL-2* promoter i-motif, further indicating its strong binding affinity to *BCL-2* promoter i-motif compared with other compounds.

### Compound A22 could selectively bind to and stabilize *BCL-2* promoter i-motif

In order to know if **A22** could specifically bind to *BCL-2* promoter i-motif, we performed SPR, MST, and ITC experiments to study the binding affinity of **A22** to *BCL-2* promoter G-quadruplex and other DNA structures. The *K*_D_ values for binding of **A22** to *BCL-2* G-quadruplex (estimated to be 48.9 μM), duplex DNA (>50 μM), *VEGF* promoter i-motif (36.4 μM), *C-KIT* promoter i-motif (34.5 μM) and *C-MYC* promoter i-motif (11.95 μM) were found to be significantly higher than that for binding of **A22** to *BCL-2* promoter i-motif (3.56 μM) in SPR assay, as shown in Supplementary Figure S1. The *K*_D_ values for binding of **A22** to *BCL-2* promoter i-motif were determined to be 4.69 and 5.52 μM by using MST and ITC (Supplementary Figure S1), respectively. To further understand the interaction of **A22** to other gene i-motifs, we used fluorescence titration assay to determine their binding affinities. As shown in Supplementary Figure S1, **A22** could induce *BCL-2* promoter i-motif formation by decreasing the fluorescence intensity of *BCL-2* promoter C-rich oligonucleotide, which was labeled with FAM at 5′-end and TAMRA at 3′-end. It has been known that the fluorescence of FAM and TAMRA double-labeled C-rich oligonucleotide is quenched when folding into i-motif structure. In comparison, the fluorescence intensities of other gene promoter i-motifs were not significantly affected upon addition of **A22**. These results are consistent with our TO replacement result. The relative replacement ratio of **A22** for *BCL-2* promoter i-motif was 52%, comparing to 10% for *BCL-2* promoter G-quadruplex, 12% for hairpin, and 6% for GC-rich duplex structures (Supplementary Figure S2). All these results showed that **A22** could specifically bind to *BCL-2* promoter i-motif.

In order to further confirm the binding between **A22** and *BCL-2* promoter i-motif, we carried out CD, ^1^H NMR, and ESI-MS experiments to study their interactions. Our CD spectra showed an increase of the i-motif band upon addition of 20 equivalents of **A22** at pH 5.5 and pH 6.2 (Supplementary Figure S2). Our ^1^H NMR spectra for the imino proton peaks of *BCL-2* promoter i-motif are shown in Supplementary Figure S3A. At pH 5.5, the imino proton peaks at 15−16 ppm for hemi-protonated C−C+ base pairs in i-motif structures were increased upon **A22** titration at 30, 40, 50, 60°C, indicating that **A22** could induce the formation of *BCL-2* promoter i-motif. When the temperature rose to 60°C, the characteristic peak of *BCL-2* promoter i-motif disappeared. Upon addition of **A22**, a peak appeared at 15–16 ppm in imino proton region, suggesting that the compound could induce the formation of i-motif and stabilize the secondary structure at high temperature. Supplementary Figure S3B showed similar results at pH 6.0. **A22** could induce the formation of i-motif at 30, 40 and 50°C. At 50°C, the free DNA showed no signal in the imino proton region, and the addition of **A22** could induce i-motif formation with peak appeared at 15–16 ppm, and stabilize the i-motif at high temperature.

**Figure 2.**
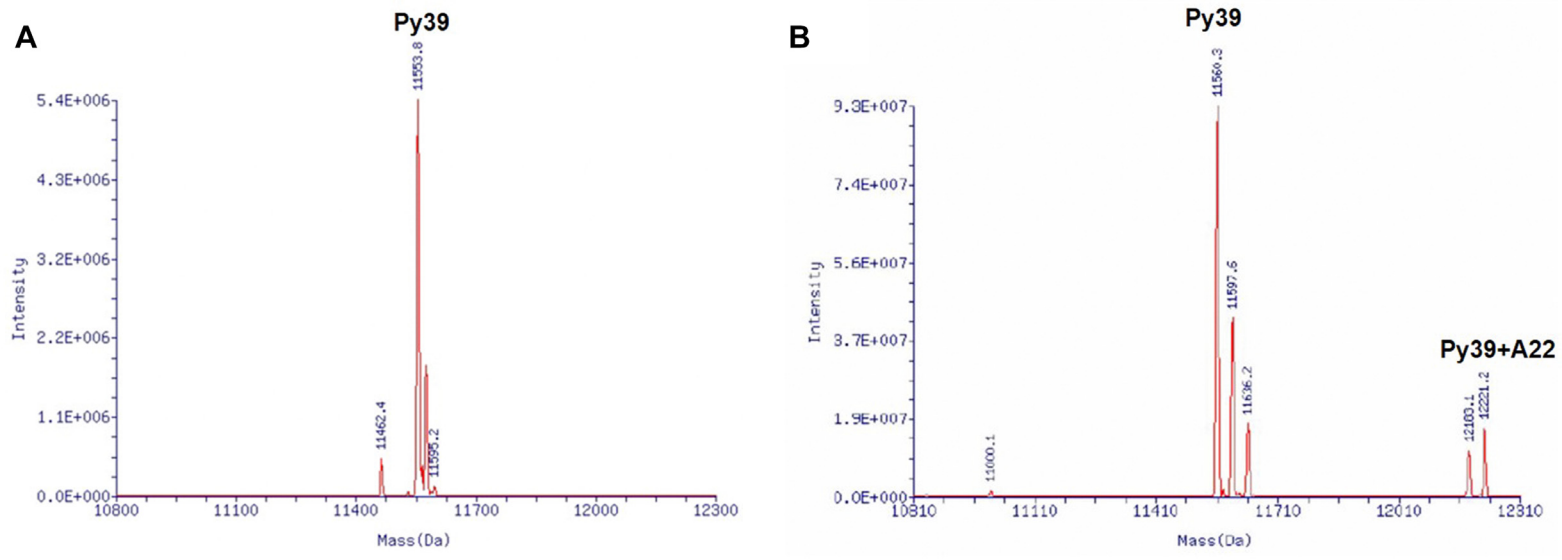
ESI-MS spectra of oligomer py39 with or without addition of **A22** at pH 6.0. (**A**) Oligomer py39 alone at pH 6.0. Py39 was annealed in 1× BPES buffer at pH 6.0. (**B**) Oligomer py39 with addition of **A22** at pH 6.0. The mass of 12 221 Da corresponding to py39–**A22** adduct was detected. All the experiments were repeated for three times.

In ESI-MS experiments (Supplementary Figure S3), py39 was incubated with **A22** at pH 7.0, and a peak of 12,186 Da for py39-**A22** adduct appeared on mass spectrum, indicating that **A22** could tightly bind to py39. The similar result was more obviously observed at pH 6.0, as shown in Figure 2A and B. These results strongly supported that **A22** could bind to and induce the formation of *BCL-2* promoter i-motif and stabilize the structure even under near physiological pH conditions.

An ideal i-motif ligand should possess two essential features: high i-motif binding specificity and high i-motif stabilizing ability. In order to know whether **A22** could stabilize *BCL-2* promoter i-motif, we carried out circular dichroism (CD) melting and FRET melting studies. In the presence of **A22**, the CD melting temperature of py39 increased for 9.63°C (Figure 1C). In contrast, **A22** had much weaker effect with *ΔTm* values ranged from 0 to 4.26°C for *BCL-2* promoter G-quadruplex (Figure 1D) and other genes (*C-KIT, KRAS, VEGF, C-MYC*) promoter i-motifs (Supplementary Figure S2). In our FRET melting experiment, **A22** could increase the melting temperature of *BCL-2* promoter i-motif for 9.15°C at 4 μM concentration. Meanwhile, **A22** had much weaker effect on melting temperatures of other gene promoter i-motifs (Supplementary Figure S2). These results showed that **A22** could specifically bind to and stabilize *BCL-2* promoter i-motif.

### A22 could up-regulate *BCL-2* gene transcription and translation in a dose-dependent manner in HepG2 cell line

**Figure 3.**
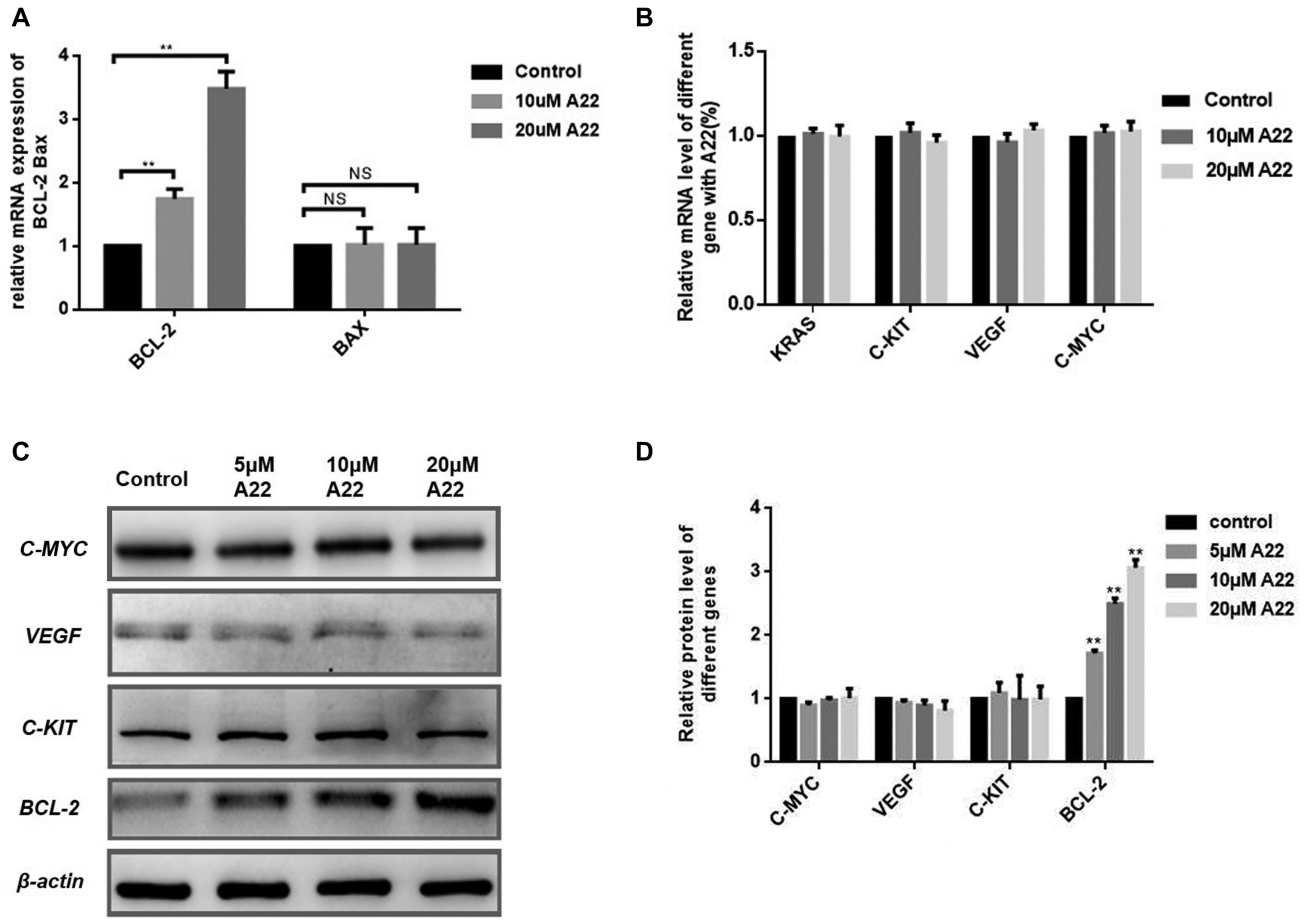
Effect of **A22** on gene transcription and translation in HepG2 cells. The mRNA levels of BCL-2 and BAX (**A**), as well as C-KIT, KRAS, C-MYC and VEGF (**B**) in HepG2 cells were analyzed by using qRT-PCR after incubation with increasing concentration of **A22** for 12 h. (**C**) Effects of **A22** on protein expressions of C-MYC, VEGF, C-KIT and BCL-2 in the presence or absence of increasing concentration of **A22** for 24 h, which were quantitatively analyzed (**D**). All the experiments were repeated for three times. The data are expressed as the mean ± SEM: (*) *P <* 0.05, (**) *P <* 0.01, significantly different from the control.

Since **A22** could efficiently bind to *BCL-2* promoter i-motif, we then studied whether **A22** could affect *BCL-2* gene transcription and translation in HepG2 cells, which were analyzed in lip-induced and steatotic cell models. **A22** was almost non-toxic to most cell lines, with its IC_50_ value determined to be more than 100 μM for HepG2 cells after 24 h incubation, as shown in Supplementary Table S4. HepG2 cells were treated with increasing concentrations of **A22**, as shown in Figure 3A. **A22** was found to significantly up-regulate *BCL-2* transcription in HepG2 cells in a dose-dependent manner. In order to know its selectivity, we studied whether **A22** could affect transcription and translation of some other well-known genes, since these genes also have i-motif structure on their gene promoters. **A22** was found to have no significant effect on transcription of *BAX*, *C-KIT*, *KRAS, C-MYC* and *VEGF* (Figure 3B). *BCL-2* translation was also up-regulated in HepG2 cells upon treatment with **A22**, with its protein levels increased in a dose-dependent manner as shown in Figure 3C and D. We also studied the selectivity of **A22** for its effect on translation of *C-KIT*, *C-MYC* and *VEGF*. As shown in Figure 3C and D, our results showed that **A22** had no significant effect on expressions of these genes.

In order to understand the mechanism for *BCL-2* gene up-regulation induced by **A22**, we carried out some experiments to study the binding interactions between *BCL-2* promoter i-motif and transcriptional factor in the presence and absence of **A22**. It has been reported that protein hnRNP LL as a transcriptional factor can recognize and bind to *BCL-2* promoter i-motif forming a transcriptional complex to activate gene transcription (13,14). We studied this interaction in the presence and absence of **A22** at extracellular level by using MST and ELISA experiments. Our purified protein hnRNP LL was half-diluted 15 times in PBS buffer (pH 7.4, 0.05% Tween-20) for binding studies with *BCL-2* promoter i-motif, and their binding constant *K*_D_ value was determined to be 26.6 μM by using MST. Upon addition of 20 equivalent of **A22**, their *K*_D_ value was significantly decreased to 2.32 μM, indicating that binding of **A22** to *BCL-2* promoter i-motif could greatly increase the binding of *BCL-2* promoter i-motif with hnRNP LL (Supplementary Figure S4A and B). Our binding study result from ELISA experiment was consistent with our MST experimental result, with their binding affinity increased significantly upon addition of **A22**, as shown in Supplementary Figure S4C.

The binding interactions between *BCL-2* promoter i-motif and transcriptional factor hnRNP LL in the presence and absence of **A22** was further studied in HepG2 cells. DNA pull-down experiment was performed to investigate their binding interactions. The biotin-labeled DNA was fastened on magnetic beads and incubated with cell lysis solution for 1 h in the presence or absence of **A22**. The amount of hnRNP LL bound with magnetic beads was analyzed by using western Blot. As shown in Supplementary Figure S4D, **A22** could significantly increase the binding of cellular hnRNP LL to *BCL-2* promoter i-motif with increased efficiency of 84.3% in the presence of 20 equivalent of **A22**, which showed that binding of **A22** to *BCL-2* promoter i-motif could promote its binding with hnRNP LL from HepG2 cells.

Chromatin Immunoprecipitation (CHIP) experiment was also carried out to study whether **A22** could affect the binding of *BCL-2* promoter i-motif with hnRNP LL in HepG2 cells. After 24 h incubation of HepG2 cells with or without **A22** at 37°C cell incubator, protein–DNA complexes of HepG2 cells were crosslinked with formaldehyde. Cells were lysed and analyzed with hnRNP LL specific antibody, and DNAs were purified and quantified with qPCR. Our result showed that *BCL-2* promoter i-motif significantly recruited hnRNP LL in the presence of **A22** in a dose-dependent manner, as shown in Supplementary Figure S4E. These results showed that **A22** could promote the binding of *BCL-2* promoter i-motif to hnRNP LL. This indicated that **A22** might up-regulate *BCL-2* gene expression through its binding to *BCL-2* P1 promoter i-motif which then recruited transcriptional factor hnRNP LL forming a complex to activate transcription.

### A22 showed dose-dependent anti-apoptosis in lipid-induced apoptosis cell model and improved hepatocyte function of glucose and lipid metabolism

**Figure 4.**
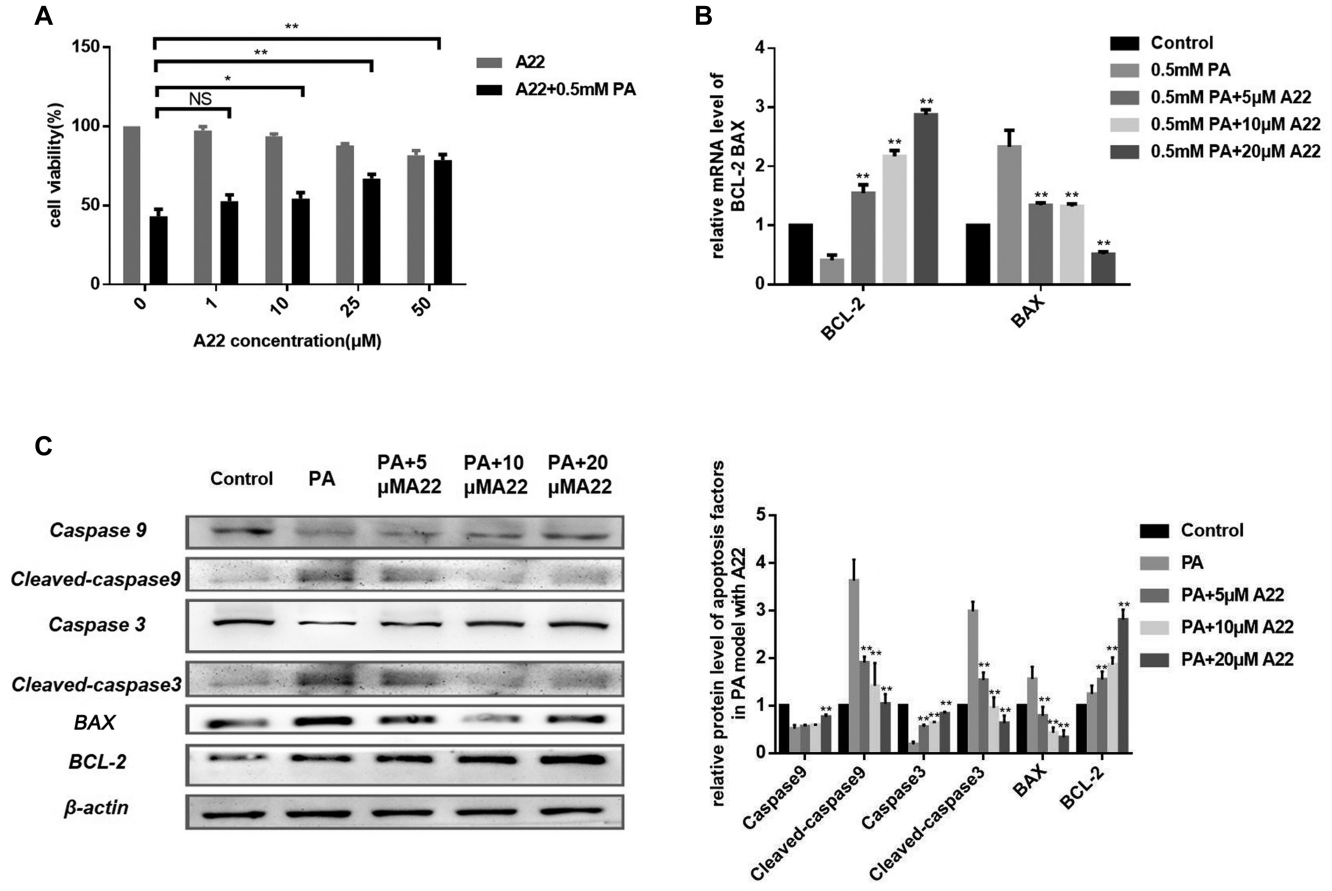
Effect of **A22** on anti-apoptosis in 0.5 mM palmitic acid oil (PA) induced cell model. (**A**) Effect of **A22** on cell viability for anti-apoptotic protective effect. (**B**) Effect of **A22** on transcription of BCL-2 and BAX with measurement of mRNA levels. (**C**) Effect of **A22** on protein expressions related with apoptosis (left), which were quantitatively analyzed (right). All the experiments were repeated for three times. The data are expressed as the mean ± SEM: (*) *P <* 0.05, (**) *P <* 0.01, significantly different from the control.

Excessive hepatocyte apoptosis has been found to be related with NASH (28), and therefore anti-apoptosis of hepatocyte could block pathological progression of NASH. Since **A22** could up-regulate *BCL-2* expression that is related with anti-apoptosis, we further studied its anti-apoptosis effect on lipid-induced hepatocyte apoptosis cell model. 0.5 mM palmitic acid oil (PA) induced hepatocyte apoptosis cell model was used. As shown in Figure 4A, after treatment with 0.5 mM palmitic acid oil (PA) for 24 h, cell viability was reduced to 43%, which was used as a lipid-induced apoptosis model. Interestingly, cell viability was improved by 11% (*P* = 0.029), 24% (*P* = 0.001), and 35% (*P* = 0.0004) upon addition of 10, 20 and 50 μM **A22**. We measured mRNA and protein levels of BCL-2 and BAX in apoptotic cell models. As shown in Figure 4B, the relative mRNA level of anti-apoptosis factor *BCL-2* decreased in PA model. With **A22** addition, *BCL-2* mRNA levels increased by 54% (*P* = 0.022), 128% (*P* = 0.003), and 190% (*P* = 0.001) upon addition of 5, 10 and 20 μM **A22**. The pro-apoptotic factor *BAX* showed an opposite trend to *BCL-2*. Meanwhile, the protein level of BCL-2 was also significantly improved compared with the PA group (Figure 4C). FITC Annexin V/PI apoptosis detection method was used for the study on 0.5 mM palmitic acid oil (PA) induced hepatocyte apoptosis cell model without or with increasing concentration of **A22** treatment for 24h, and collected cells were analyzed by using flow cytometry. As shown in Supplementary Figure S5A and C, in contrast to the control cells with an apoptotic ratio of 5%, ratio of apoptotic cells in PA induced group was significantly increased to 24.6%. Upon addition of 20 μM **A22**, the apoptosis ratio was reduced to 16% (*P* = 0.019), and **A22** displayed apparent anti-apoptotic activity in a dose-dependent manner. The mitochondrial membrane potential assay which is an indicator of early apoptosis was also performed by using JC-1 fluorescence probe (Supplementary Figure S5E). The membrane-permeant JC-1 dye is widely used in apoptosis studies to monitor mitochondrial health. Mitochondrial depolarization in early apoptosis is indicated by a decrease in the red/green fluorescence intensity ratio. As the concentration of **A22** increased, the red/green fluorescence intensity ratio increased, indicating that **A22** could decrease the occurrence of early apoptosis. In addition, we also found that **A22** could reduce general oxidative stress in 0.5 mM PA-induced apoptosis model with H2DCFDA probe and protect mitochondria in living cells by the Mitortracker deep red probe (Supplementary Figure S5G). Then we studied caspases of apoptosis signaling pathways in PA-induced models. It was found that **A22** could effectively decrease PA-induced activation of apoptosis related proteins in a dose-dependent manner, with decreased cleaved caspase 3 (C-Caspase 3) and 9 (C-Caspase 9) (Figure 4C). These results all showed that **A22** could alleviate apoptosis and improve cell survival in PA-induced apoptosis model.

We also examined whether **A22** could affect glucose and lipid metabolism of HepG2 cells after up-regulating *BCL-2* gene expression against apoptosis. As shown in Supplementary Figure S5B and D, **A22** could reduce intracellular lipid deposition of 0.5 mM oleic acid (OA) induced droplet formation. The ability of hepatocyte in absorbing glucose in insulin resistant model was also studied by using 2-NBDG (2-(*N*-(7-nitrobenz-2-oxa-1,3-diazol-4-yl)amino)-2-deoxyglucose). HepG2 cells were induced to IR (insulin resistance) with insulin (10^−6^ M) treatment for 24 h, and effect of **A22** on cellular glucose intake was measured by using confocal imaging. Our results (Supplementary Figure S5F) showed that **A22** could significantly improve hepatocyte capacity of glucose intake, compared with positive drug Metformin (1 mM). In conclusion, our results were well consistent, which all showed that **A22** could improve hepatocyte function through anti-apoptosis in lipid-induced cell models.

### A22 could ameliorate apoptosis followed with attenuating hepatic injury and steatosis for high-fat diet-fed mice model

**Figure 5.**
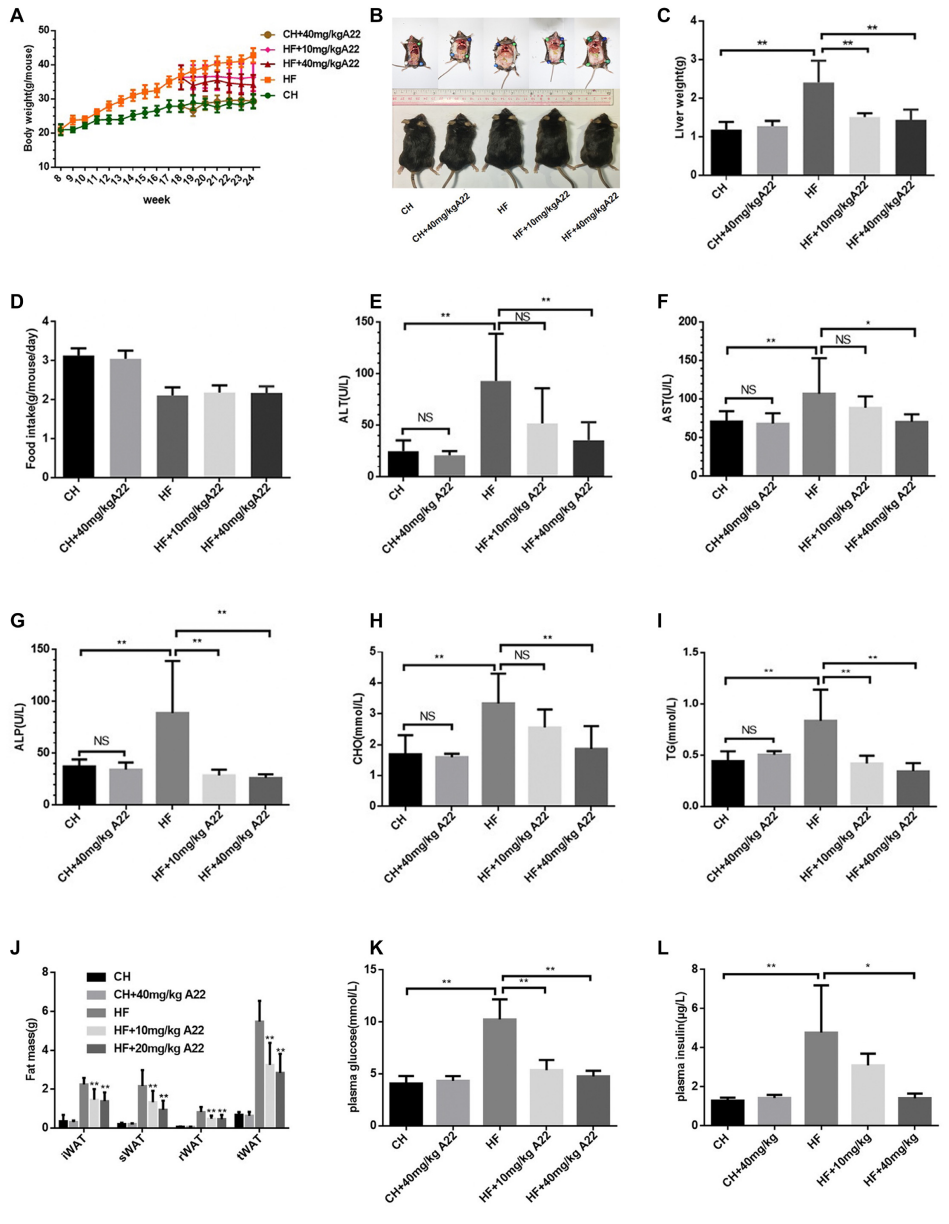
Effect of **A22** on various body parameters in NAFLD/NASH mice model. Male adult C57/B6J mice were fed chow (CH) or high-fat (HF) diet for 16 weeks, and **A22** was administrated in the last 6 weeks to two groups (10 and 40 mg/kg. i.p. every other day). Body/tissue weights and serum as well as liver parameters were analyzed in the end of the experiment after 8 h of fasting, and the data were obtained in following category: (**A**) body weight, (**B**) representative individuals of each group, (**C**) liver weight, (**D**) food-intake, (**E**) alanine aminotransferase (ALT), (**F**) aspartate aminotransferase (AST), (**G**) alkaline phosphatase (ALP), (**H**) total cholesterol, (**I**) triglyceride, (**J**) fat mass, (**K**) plasma glucose and (**L**) plasma insulin. Data are statistically analyzed as means ± SEM, and each circle or column indicated one group (*N* = 8 mice/group). * means *P <* 0.05, versus HF control mice; ** means *P <* 0.01, versus HF control mice.

In order to evaluate whether **A22** has anti-apoptosis activity *in vivo*, we used NAFLD/NASH mice model induced by using reported methods with high-fat (HF) diet to study potential therapeutic effect of **A22** on apoptosis. Formulation of the high fat diet was list as shown in Supplementary Table S6. After 6 weeks intraperitoneal injection of increasing concentrations of **A22**, samples were then collected and analyzed. Compared with control group, serum levels of alanine aminotransferase (ALT), aspartate aminotransferase (AST), and alkaline phosphatase (ALP) in experimental group were all found to be significantly decreased in a dose-dependent manner, indicating its effect of attenuating hepatic injury (Figure 5E–G). As expected, **A22** relieved HF-induced phenotypes of metabolic syndrome indicated by reductions of body weight gain (Figure 5A and B), fat mass (Figure 5J), hyper glycemia (Figure 5K), hyperinsulinemia (Figure 5L), and high plasma level of total cholesterol (CHO) and triglyceride (TG) (Figure 5H and I). These effects were not affected by food intake (Figure 5D).

**Figure 6.**
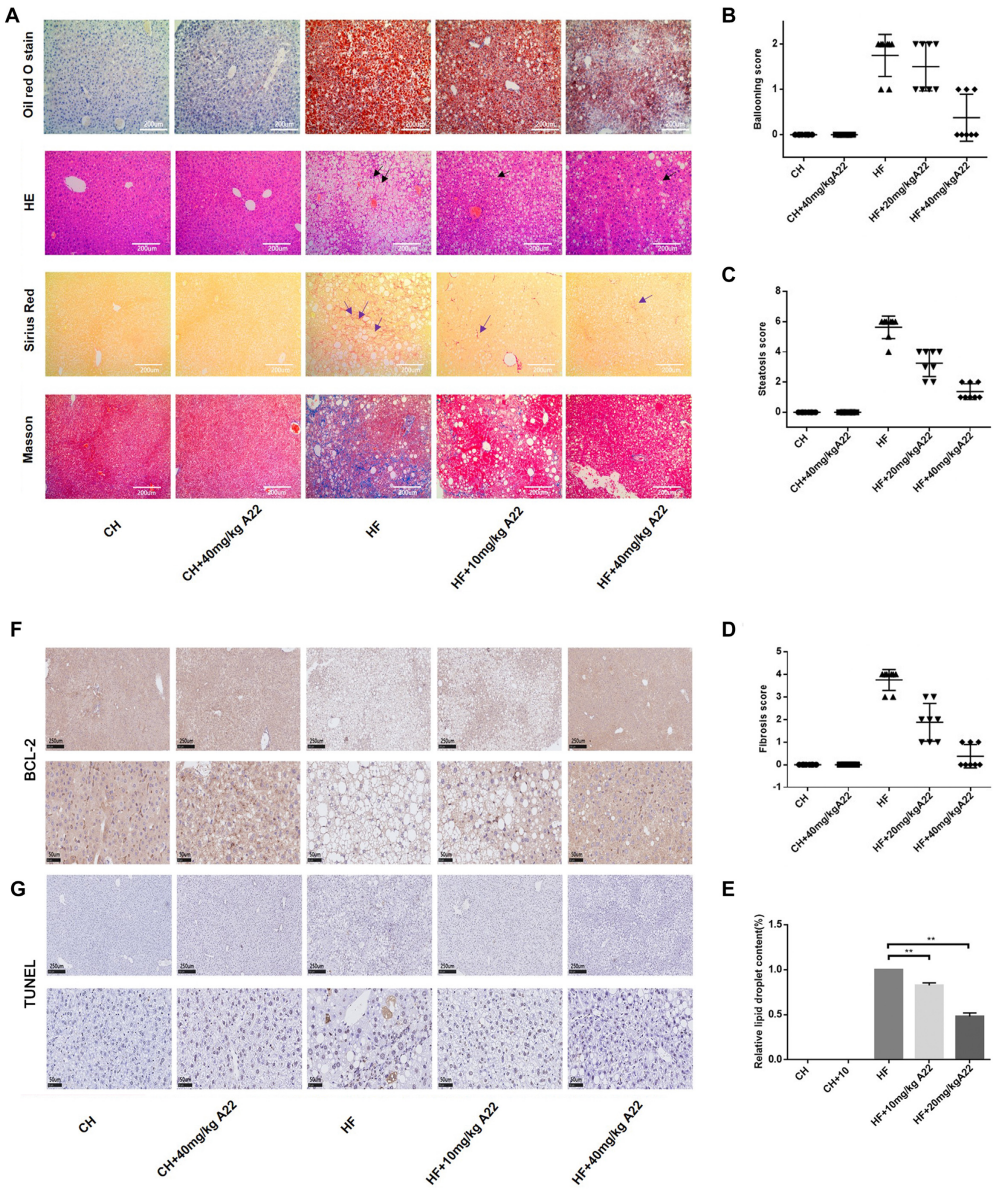
Effect of **A22** on alleviating morphological changes of mice livers. (**A**) Representative images of livers (× 100 magnification) with oil red O staining, H&E staining, Sirius Red staining, and masson staining. (**B–D**) Ballooning (blue arrow indicated), hepatic steatosis (black arrow indicated), and fibrosis (blue arrow indicated) scores were obtained according to the NAFLD Activity Score (NAS) System as described in Methods. (**E**) Relative lipid droplet contents were determined. (**F**) Expressions of BCL-2 in livers of each group were determined by using immunohistochemistry. (**G**) Liver apoptosis was determined by using a TUNEL assay in situ. Representative images were captured with stained black dots indicated in the image. Data were statistically analyzed as means ± SEM, and each circle indicated one group (*N* = 8 mice/group). *P< 0.05, versus HF control mice; **P< 0.01, versus HF control group.

**Figure 7.**
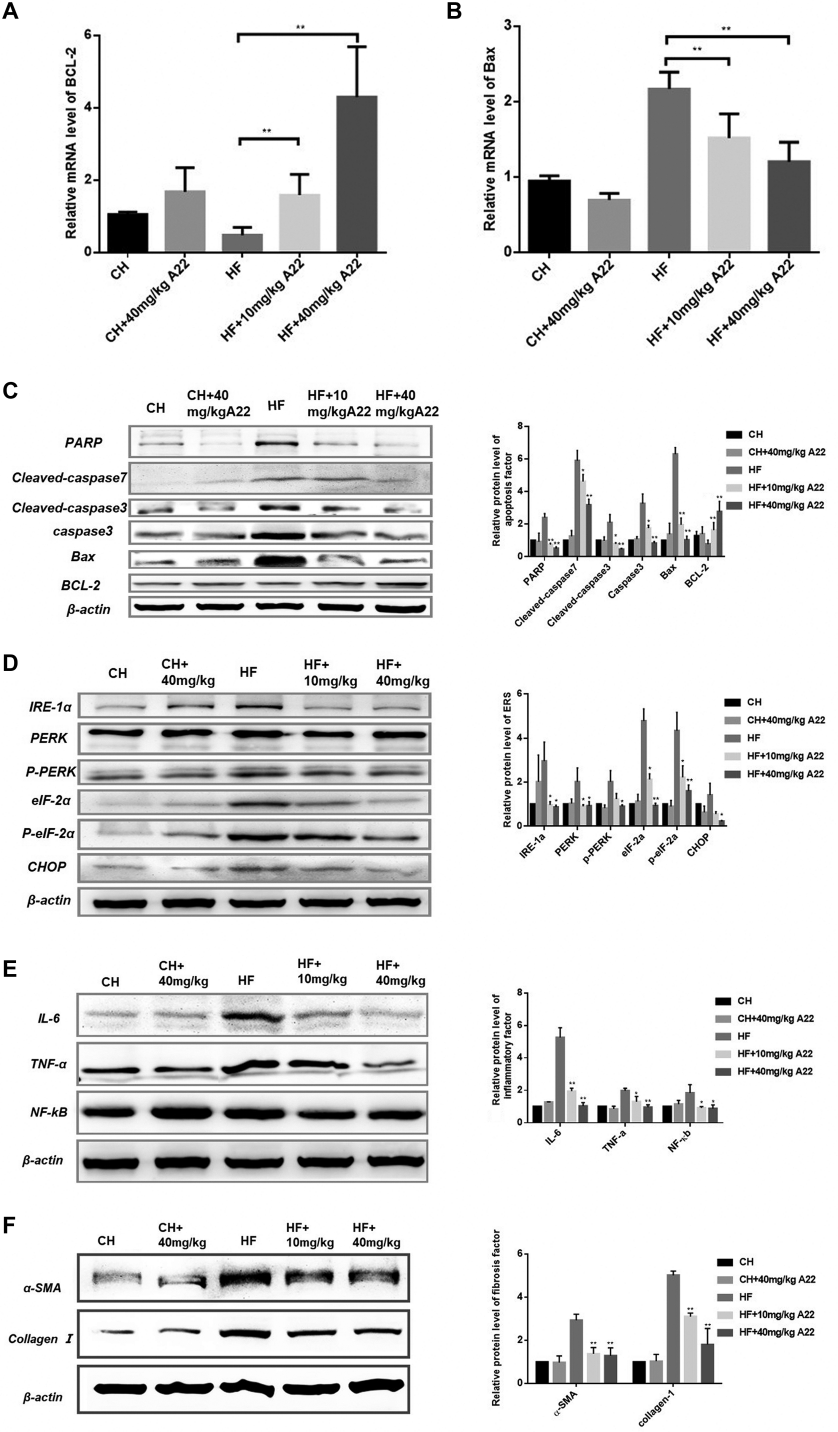
Effect of **A22** on ameliorating apoptosis, ER stress, inflammation, metabolic syndrome, and fibrogenesis in HF diet-fed mice. (**A**) Effect of **A22** on BCL-2 gene transcription. (**B**) Effect of **A22** on BAX gene transcription. (**C**) Effect of **A22** on expressions of apoptosis-related proteins in liver. The extracted proteins from the liver were immunoblotted with specific antibodies, and quantified based on the loading control of ACTIN. (**D**) Effect of **A22** on ER stress. The UPR proteins (IRE-1α, PERK, elF-2α and CHOP) were analyzed by using western Blot. (**E**) Effect of **A22** on expressions of inflammatory factors. (**F**) Effect of **A22** on expressions of fibrogenic proteins. Data were statistically analyzed as means ± SEM, and each column indicated one group (*N* = 8 mice/group). *P< 0.05, versus HF control mice; **P< 0.01, versus HF control group (*N* = 8 mice /group).

Interestingly, **A22** administration almost corrected all the phenotypes of NAFLD/NASH induced by HF diet, namely histological steatosis and hepatocyte ballooning (Figure 6A), compared with normal levels of chow (CH) diet-fed mice. Ballooning (blue arrow indicated), hepatic steatosis (black arrow indicated), and fibrosis (blue arrow indicated) scores were obtained according to the NAFLD Activity Score (NAS) System as shown in Figure 6B–D respectively. Relative lipid droplet contents were determined as shown in Figure 6E. **A22** also decreased liver weight of HF diet-fed mice (Figure 5C). Then we examined transcription of *BCL-2* gene and expression of apoptosis-related proteins in mice liver samples. For CH fed mice with 40 mg/kg **A22** group, their *BCL-2* and *BAX* transcriptions did not have significant difference from CH control group. However, significant changes of *BCL-2* and *BAX* transcriptions occurred for HF diet-induced NAFLD/NASH model, as shown in Figure 7A and B. Their mRNA levels were analyzed by using qRT-PCR. The chronic HF diet-fed mice had increased hepatocellular apoptosis in the liver, as indicated by their increased expressions of cleaved-caspase 3, PARP, BAX, and decreased expression of BCL-2 (Figure 7C). **A22** treatment significantly affected the levels of apoptotic markers and increased BCL-2 expression. The similar results were also obtained for immunohistochemical stain of BCL-2 (Figure 6F) and cleaved-caspase 3 (Supplementary Figure S7A and B). Our above experimental results were consistent with the dose-dependent reduction of hepatocyte apoptosis of **A22** detected in Tunnel experiment (Figure 6G), which could directly reflect the apoptosis of hepatocytes in liver tissue.

It has been reported that increased hepatocyte apoptosis is a pivotal mechanism contributing to inflammation and fibrogenesis of NASH (29). Hepatocyte apoptosis can be activated by many causes including lipid toxicity (30), unresolved endoplasmic reticulum (ER) stress (31) and oxidative stress (32). ER stress has been reported to be increased during NAFLD/NASH, which can promote lipid synthesis and inflammation (30). Proteins PRKR-like endoplasmic reticulum kinase (PERK) and eukaryotic translation initiation factor 2 alpha (eIF-2α) can initiate unfolded protein response (UPR) canonical pathway to increase the ER stress, and UPR accumulation can result in activation of apoptosis. Therefore, we examined potential therapeutic effect of **A22** on hepatocyte ER stress. Our results (Figure 7D) showed that **A22** reduced expression levels of inositol-requiring enzyme 1α (IRE 1α), phosphorylated PERK, phosphorylated eIF-2α, and C/EBP homologous protein (CHOP) all in a dose-dependent manner. These indicated that **A22** could ameliorate apoptosis partially through its effect on ER stress.

In order to know whether **A22** has anti-inflammatory and anti-fibrosis effects, we studied its effects on HF diet-fed mice liver. Our results showed that **A22** treatment significantly inhibited HF diet-induced macrophage aggregation, which were analyzed with specific antigen CD11b (for monocyte-derived macrophages) (Supplementary Figure S7A and C) and CD45 (for lymphocyte) (Supplementary Figure S7A and D). These were associated with decreased protein levels of inflammatory cytokines NF-κB, TNF-α, IL-6 of the liver (Figure 7E). Besides, **A22** also showed significant anti-fibrosis effect by reducing protein expressions of α-SMA and Collagen I (Figure 7F), as well as reversing the fibrosis stained with masson and Sirius Red (Figure 6A and D). The above results indicated that **A22** could effectively alleviate not only hepatocyte apoptosis induced by high-fat diet, but also pathological changes including balloon-like degeneration, inflammatory response, fibrosis, and hepatocyte damage.

In order to evaluate whether **A22** could affect the ability of liver in metabolizing sugar *in vivo*, intraperitoneal glucose tolerance test (IPGTT) and intraperitoneal insulin tolerance test (IPITT) were performed on HF diet-fed mice with 4 weeks of **A22** treatment. Compared to CH diet-fed mice group, the Area Under Curve parameters (AUC_glucose_, AUC_ITT_) and Home-IR of HF diet-fed mice were apparently increased (*P* < 0.0001, *P* < 0.0001), indicating increased glucose tolerance. With addition of **A22**, the AUC value and insulin resistance index (HOME-IR) decreased in a dose-dependent manner (Supplementary Figure S6), which indicated fast glucose disappearance and therefore improved glucose tolerance. These results showed that **A22** could bind to and stabilize *BCL-2* promoter i-motif resulting in up-regulation of *BCL-2* expression, which could become a promising approach for anti-apoptosis based on our study in NAFLD/NASH mice model *in vivo*. **A22** could become a multi-functional lead compound for further development for NAFLD/NASH treatment through improving hepatic functions including reducing lipid accumulation, glucose metabolism, anti-inflammation, and inhibition of fibrosis.

## DISCUSSION

Thus far, lifestyle intervention and weight loss are main treatments for NAFLD/NASH approved by FDA. Although some agents, such as obeticholic acid, have been proved to alleviate NASH in phase 3 clinical trials (33), no pharmacotherapy is clinically available. It has been shown that activation of caspases (34), BCL-2 family proteins (35), and c-Jun *N*-terminal kinase (36) induced hepatocyte apoptosis, which play important roles in activation of NAFLD/NASH. Apoptotic hepatocytes can stimulate hepatic stellate cells and immune cells (37–40) to produce inflammatory factors and cytokines in the progression of fibrosis in liver. Disorder of glucose (41) and lipid metabolism (42) associated with NAFLD/NASH patients can accelerate the progress. The activation of reactive oxygen species (43,44), oxidative stress, and endoplasmic reticulum stress (45) are also involved. The pathogenesis of steatohepatitis contains a complicated reprogrammed molecular network. Therefore, it is desirable to develop new strategy for NASH treatment by targeting a key factor involved in pathogenic pathways to produce multifunctional and synergistic effects. The anti-apoptotic factor BCL-2 seems to be an ideal target, and its inhibition or down-regulation can trigger mitochondrial pathway of apoptosis and initiate caspase cascade reaction. It has been shown that BCL-2 expression is significantly low for NASH patients (10,46), suggesting that the pathological progression of NASH was negatively correlated with BCL-2 expression level.

After extensive screening, we discovered acridone derivative **A22** as a promising suppressor of steatohepatitis by stabilizing *BCL-2* gene promoter i-motif followed with up-regulation of anti-apoptosis factor BCL-2. **A22** could significantly improve cellular and animal models in aspects of insulin resistance and lipid deposition after alleviating hepatocyte apoptosis. From a clinical perspective, our study highly implicated future development of small molecules with *BCL-2* gene promoter i-motif as target for disrupting hepatocyte apoptosis as a promising avenue for NASH therapy. Our results showed that **A22** could induce formation of *BCL-2* promoter i-motif under near-neutral conditions. **A22** could bind to *BCL-2* promoter i-motif with good selectivity, since it had no significant binding to the i-motifs from other tested genes such as *C-KIT* and *VEGF*. The effect of **A22** on gene expression in cells also showed that **A22** could up-regulate BCL-2 expression without significant effect on other gene expressions. Compared with other complex methods for up-regulating gene expressions, our approach of using small molecules with gene promoter i-motif as target is relatively more practical.

Notably, BCL-2 not only controls classical caspase cascade, but also plays an important role in mitochondrial pathway in relation to apoptosis. The interference of BCL-2 associated apoptotic signaling pathway in hepatocyte can affect hepatocyte function, inflammatory response, and hepatic fibrosis. Our results showed that selective up-regulation of BCL-2 expression in hepatocyte not only increased mitochondrial membrane potential (red/green fluorescence intensity ratio) in early apoptosis, but also blocked caspase cascade reaction. By blocking hepatocyte apoptosis, **A22** effectively gained function of hepatocytes, offset occurrence of pathological changes in NAFLD/NASH, and relieved steatohepatitis.

The expression levels of BCL-2 in liver and serum have been found to be down-regulated in NAFLD/NASH patients, suggesting that BCL-2 play an important role in hepatocyte apoptosis in NAFLD/NASH. By up-regulating BCL-2 expression, **A22** showed apparent anti-apoptosis effect in many ways. The mitochondrial pathway of apoptosis is triggered by losing the integrity of mitochondrial outer membrane, which allows the release of proapoptotic factors (e.g., cytochrome c) from the mitochondria into the cytosol. This process is controlled by BCL-2. On the other hand, activation of caspase family members which are triggered by BCL-2 is also a critical mechanism of apoptosis. Our results showed that by up-regulating *BCL-2*, **A22** could increase the red/green fluorescence intensity ratio (Supplementary Figure S4D) and inhibit the activation of caspase 3 and 9 (Figure 4C), indicating its possible multi-functional anti-apoptosis mechanisms.

NAFLD/NASH is characterized by liver injury, inflammation and fibrosis of pathological progression. Progression of hepatocyte apoptosis results in cell function impairment, liver inflammation and oxidative stress. Since **A22** could act as an anti-apoptosis molecule for NAFLD/NASH, we studied whether it could offset occurrence of pathological changes. We found that **A22** could effectively reduce liver damage as indicated by reductions of serum AST, ALT and ALP, and liver morphology tissue damage compared with HF diet-fed group. The accumulation of inflammatory factors occurred in HF diet-fed mice livers, characterized by up-regulation of inflammatory factors and accumulation of mononuclear macrophages and lymphocytes. **A22** treatment effectively reduced expressions of inflammatory factors and attenuated inflammatory cell cluster. Liver fibrosis was also reversed in HF diet-fed mice after **A22** addition through down-regulation of corresponding proteins such as collagen 1 and α-SMA involved in the development of fibrosis as well as collagen fibers aggression.

Liver is a key organ in pathogenesis of metabolic syndrome, which controls a vast majority of glucolipid metabolism. By blocking hepatocyte apoptosis, **A22** could effectively alleviate the symptoms associated with metabolic syndrome including weight loss, glucose metabolism (IPGTT and IPITT), serum lipid levels, and insulin resistance index (HOME-IR) in mice. In addition, **A22** reduced TG accumulation in the liver of HF diet-fed mice and cultured cells incubated with fatty acids. These strongly supported that increased expression of BCL-2 is significantly beneficial to hepatocyte function. Liver plays an important role in metabolism and utilization of glucose and lipid. We examined hepatocyte function of absorbing glucose both in lip-induced cells and animal models. The insulin resistance was significantly improved by **A22** administration in a dose-dependent manner, as indicated by increase of 2-NBDG intake in HepG2 cells. The ability of glucose metabolism (IPGTT, IPITT, HOME-IR) after **A22** administration *in vivo* was also improved. Interestingly, the accompanying weight loss was also observed. Overall, the present study demonstrated that **A22** could up-regulate BCL-2 expression by targeting *BCL-2* promoter i-motif, which showed significant anti-apoptosis effect to offset hepatocyte impairment, inflammation, and fibrogenesis in pathological progression of NAFLD/NASH.

## Supplementary Material

gkaa615_Supplemental_File
