# Supplementary material for: Upregulation of BCL-2 by acridone derivative through gene promoter i-motif for alleviating liver damage of NAFLD/NASH
Source: Nucleic Acids Res. 2020 Jul 25;48(15):8255–68. doi: 10.1093/nar/gkaa615 (PMC7470982; doi:10.1093/nar/gkaa615)
Supplement: gkaa615_Supplemental_File [file gkaa615_supplemental_file.pdf]

Supplementary Material for

Upregulation of *BCL-2* by Acridone Derivative through Gene Promoter i-motif for Alleviating Liver Damage of NAFLD/NASH

Xiaoya Li, Jing Wang, Xue Gong, Meiling Zhang, Shuangshuang Kang, Bing Shu, Zuzhuang Wei, Zhi-Shu Huang, and Ding Li*

School of Pharmaceutical Sciences, Sun Yat-sen University, Guangzhou University City, 132 Wai huan East Road, Guangzhou 510006, P. R. China

* Corresponding author：Ding Li, Tel: 8620 3994 3058; E-mail: liding@mail.sysu.edu.cn

Table of Contents

Table S1. The oligonucleotides used in the present study S3

Table S2. Equilibrium binding constants (K_D) determined by using SPR S5

Table S3. Relative TO displacement ratio (%) of compounds screened in SPR experiment S7

Table S4. IC_{50} values (μM) of **A22** to various cell lines (24 h) measured by using MTT S7

Table S5. Primers sequence information S8

Table S6. Formulation of the high fat diet S9

Table S7. The qPCR raw data from the real-time RT-PCR assay in cells and animal samples S10

Figure S1. Biophysical experimental results for activity of acridine derivative in binding to *BCL-2* promoter i-motif and other DNA structures S12

Figure S2. Effect of acridine derivative on oncogene promoter secondary structures analyzed by using various experiments S15

Figure S3. NMR and ESI-MS spectra of py39 without or with the addition of acridine derivative S18

Figure S4. Effect of **A22** on the interaction of *BCL-2* promoter i-motif with its binding protein hnRNP LL S20

Figure S5. A22 can reduce hepatocyte apoptosis, lipid deposition, improve glucose uptake and release endoplasmic reticulum stress in vitro S22

Figure S6. Glucose Tolerance Tests of NAFLD/NASH mice S24

Figure S7. A22 inhibited lipid-induced liver apoptosis and inflammation in immunohistochemistry stain S25

Scheme I. Synthetic route for acridone **A22** and **A22-HCl** S26

MATERIAL AND METHODS S27

Table S1. The oligonucleotides used in the present study

Name	Sequence (5'-3')
py39	CAGCCCCGCTCCCGCCCCCTTCCTCCCGCGCCCGCCCCT
pu39	AGGGGCGGGCGCGGGAGGAAGGGGGCGGGAGCGGGGCTG
bio-py39	biotin-d[CAGCCCCGCTCCCGCCCCCTTCCTCCCGCGCCCGCCCCT]
bio-pu39	biotin-d[AGGGGCGGGCGCGGGAGGAAGGGGGCGGGAGCGGGGCTG]
bio-hairpin	biotin- d[TATAGCTATA-HEG-TATAGCTATA]
bio-C-KIT	biotin- d[CCCTCCTCCCAGCGCCCTCCCT]
bio-VEGF	biotin- d[GACCCCGCCCCCGGCCCGCCCCGG]
bio-py27	biotin-d[CCTTCCCCACCCTCCCCACCCTCCCCA]
Fpy39T	FAM-CAGCCCCGCTCCCGCCCCCTTCCTCCCGCGCCCGCCCCT-TAMRA
Fpu39T	FAM-AGGGGCGGGCGCGGGAGGAAGGGGGCGGGAGCGGGGCTG-TAMRA
Fpy39	FAM-CAGCCCCGCTCCCGCCCCCTTCCTCCCGCGCCCGCCCCT
Fpu39	FAM-AGGGGCGGGCGCGGGAGGAAGGGGGCGGGAGCGGGGCTG
Ftel CT	FAM-CCCTAACCCTAACCCTAACCCTAA-TAMRA
FC-KITT	FAM-CCCTCCTCCCAGCGCCCTCCCT-TAMRA
FKRAST	FAM-GCCCGGCCCCCGCTCCTCCCCCGCCGGCCCGGCCCGGCCCCCTCC TTCTCCCCG-TAMRA
FVEGFT	FAM-GACCCCGCCCCCGGCCCGCCCCGG-TAMRA
Fpy27T	FAM-CCTTCCCCACCCTCCCCACCCTCCCCA-TAMRA
hairpin	CGCGCGCGTTTTCGCGCGCG
tel C	CCCTAACCCTAACCCTAACCCTAA
C-KIT	CCCTCCTCCCAGCGCCCTCCCT
py27	CCTTCCCCACCCTCCCCACCCTCCCCA
VEGF	GACCCCGCCCCCGGCCCGCCCCGG

KRAS	GCCCGGCCCCCGCTCCTCCCCCGCCGGCCCGGCCCGGCCCCCTCCTTCT CCCCG

Table S2. Equilibrium binding constants (K_D) determined by using SPR

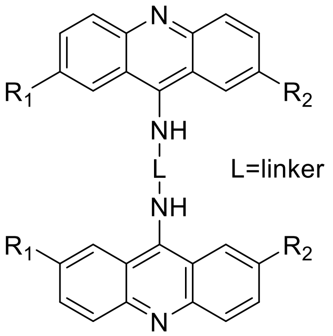

Di02 R1=-CH_3, R2=-CH_3, L=-$(CH_2)_3N(CH_3)(CH_2)_3$-
Di03 R1=-CH_3, R2=-H, L=-$(CH_2)_3N(CH_3)(CH_2)_3$-
Di04 R1=-CH_3, R2=-H, L=-$(CH_2)_3NH(CH_2)_2NH(CH_2)_3$-
Di06 R1=-CH_3, R2=-CH_3, L=-$(CH_2)_2NH(CH_2)_2$-
Di07 R1=-H, R2=-H, L=-$(CH_2)_2NH(CH_2)_2$-
Di08 R1=-H, R2=-H, L=-$(CH_2)_3N(CH_3)(CH_2)_3$-
Di09 R1=-H, R2=-H, L=-$(CH_2)_3$-
Di10 R1=-H, R2=-H, L=-$(CH_2)_3NH(CH_2)_2NH(CH_2)_3$-
Di13 R1=-CH_3, R2=-H, L=-$(CH_2)_3$-
Di15 R1=-CH_3, R2=-H, L=-$(CH_2)_7$-
Di16 R1=-CH_3, R2=-H, L=-$(CH_2)_2O(CH_2)_2O(CH_2)_2$-
Di17 R1=-CH_3, R2=-CH_3, L=-$(CH_2)_3$-
Di18 R1=-CH_3, R2=-CH_3, L=-$(CH_2)_7$-
Di19 R1=-$CH_{3,}$ R2=-H, L=-$(CH_2)_4$-
Di21 R1=-CH_3, R2=-CH_3, L=-$(CH_2)_5$-

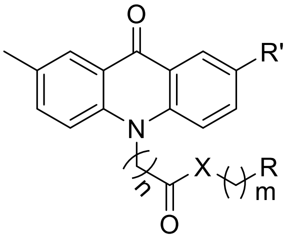

(**A3**-**A19**)

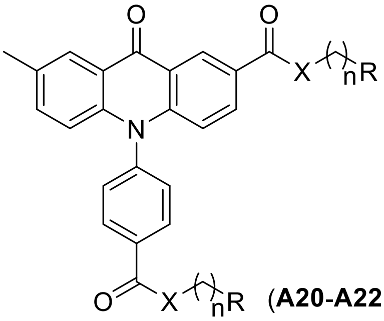

(**A20**-**A22**)

A3 X=NH n=1 m=3, R=dimethylamino-, R'=H
A4 X=NH n=1 m=2, R=morpholinyl-, R'=H
A5 X=NH n=1 m=2, R=ethylenediamine-, R'=H
A6 X=NH n=1 m=3, R=morpholinyl-, R'=H
A7 X=O n=1 m=3, R=amino-, R'=H
A8 X=NH n=2 m=2, R=(ethyl formate)-ly-, R'=H
A9 X=NH n=2 m=2, R=morpholinyl-, R'=H
A10 X=NH n=2 m=2, R=dimethylamino-, R'=H
A18 X=NH n=1 m=2, R=diethylamine-, R'=H
A19 X=O n=1 m=0, R=morpholinyl-, R'=H
A20 X=NH, n=2, R=morpholinyl-,
A21 X=NH, n=3, R=dimethylamino-
A22 X=NH, n=3, R=morpholinyl-

Compound	K_D (μM)
**Di02**	>50
**Di03**	>50
**Di04**	>50
**Di06**	>50
**Di07**	>50
**Di08**	>50
**Di09**	>50
**Di10**	>50
**Di13**	34.1
**Di15**	>50
**Di16**	>50
**Di17**	>50
**Di18**	>50
**Di19**	>50
**Di21**	.>50
**A3**	>50
**A4**	>50
**A5**	>50
**A6**	21
**A7**	13.7
**A8**	14.3
**A9**	>50
**A10**	>50
**A18**	>50
**A19**	26.5
**A20**	>50
**A21**	40.3

	**A22**	3.56
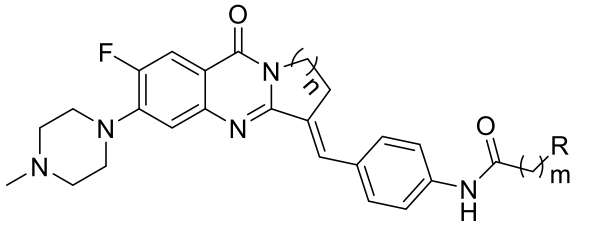<br>**7A-1** n=0, m=1, R=tetrahydropyrrolyl-<br>**7A-3** n=0, m=1, R=morpholinyl-<br>**7A-6** n=1, m=2, R=morpholinyl-<br>**7A-8** n=1, m=1, R=1- (2-hydroxyethyl) piperazine-<br>**7C-2** n=0, m=1, R=diethylamino-<br>**7C-4** n=1, m=1, R=tetrahydropyrrolyl-<br>**7C-7** n=0, m=1, R=1- (2-hydroxyethyl) piperazine-<br>**7C-9** n=0, m=2, R=tetrahydropyrrolyl-	**7A-1**	>50
	**7A-3**	>50
	**7A-6**	>50
	**7A-8**	>50
	**7C-2**	>50
	**7C-4**	>50
	**7C-7**	>50
	**7C-9**	>50
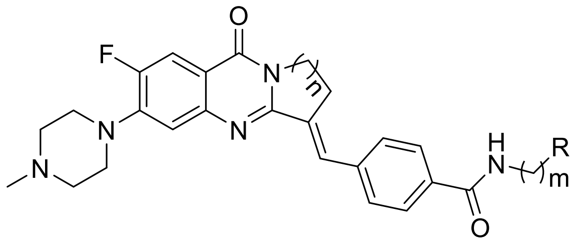<br>**7C-3** n=0, m=2, R=tetrahydropyrrolyl-<br>**7C-5** n=1, m=2, R=tetrahydropyrrolyl-<br>**7C-6** n=1, m=2, R=diethylamino-<br>**7C-8** n=1, m=2, R=piperidinyl-<br>**7D** n=1, m=2, R=dimethylamino-<br>**7D-2** n=0, m=2, R=diethylamino-<br>**7D-3** n=0, m=2, R=dimethylamino-	**7C-3**	>50
	**7C-5**	>50
	**7C-6**	>50
	**7C-8**	>50
	**7D**	>50
	**7D-2**	>50
	**7D-3**	>50
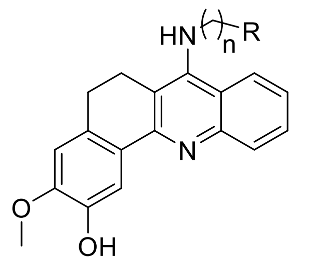 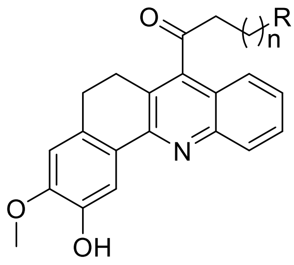<br>**1a** n=2, R=dimethylamino- **5c** n=2, R=tetrahydropyrrolyl-<br>**1b** n=2, R=diethylamino- **6b** n=3, R=diethylamino-<br>**2a** n=3, R=dimethylamino-<br>**2c** n=3, R=tetrahydropyrrolyl-<br>**2e** n=3, R=N-methylpiperazinyl-,	**1a**	>50
	**1b**	>50
	**2a**	>50
	**2c**	>50
	**2e**	>50
	**5c**	>50
	**6b**	>50
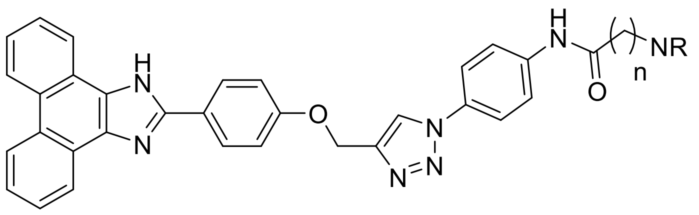	**19a**	>50
	**19d**	>50
	**19c**	>50
	**20a**	>50
	**20b**	>50

**19a** n=2, NR = pyrrolidinyl-<br>**19c** n=2, NR = piperidyl-<br>**19d** n=2, NR = hydroxyethylpiperazinyl-<br>**20a** n=1, NR = pyrrolidinyl-<br>**20b** n=1, NR = diethylamino-<br>**20c** n=1, NR = piperidyl-<br>**20d** n=1, NR = morpholinyl-<br>**20e** n=1, NR = dimethylamino-	**20c**	>50
	**20d**	>50
	**20e**	>50

Table S3. Relative TO displacement ratio (%) of compounds screened in SPR experiment

**Compound**	**Relative displacement ratio (%)**
**Di13**	5.2
**A6**	0.3
**A7**	1
**A8**	1.9
**A22**	52

Table S4. IC_{50} values (μM) of **A22** to various cell lines (24 h) measured by using MTT

cell line	**Hek293**	**Siha**	**A549**	**PANC-1**	**U87**
IC_{50} (μM)	58.71	81.38	>100	93.4	>100

cell lines	**Ins-1**	**MCF-7**	**HepG2**	**A375**	**LX-2**
IC_{50} (μM)	>100	>100	>100	>100	>100

Table S5. Primers sequence information

Spics	Gens	Forward primer (5'-3')	Reverse primer (5'-3')
	β-actin	CTGGAACGGTGAAGGTGAA	AAGGGACTTCTGTAACAACGA
	BCL-2	TGTTGTTCAAACGGGATTCA	GGCTGGGCACATTTACTGTT
	BAX	AGCGACTGATGTCCCTGTCT	CTCAGCCCATCTTCTTCCAG
	C-KIT	TATACAACCCTGGCATTATGT	TGCGAAGGAGGCTAAACCTA
huma	*VEGF*	GCTACTGCCATCCAATCGAG	CTTGGTGAGGTTTGATCCGC
n	*KRAS*	GTGGAGTATTTGATAGTGTTTAAC	TGTATCAAAGAATGGTCCTGCA
	C-MYC	AAACACAAACTTGAACAGCTAC	ATTTGAGGCAGTTTACATTATGG
	IL-6	GATGGCTGAAAAAGATGGATGC	TGGTTGGGTCAGGGGTGGTT
	NF-κB	ATGGCTTCTATGAGGCTGAG	GTTGTTGTTGGTCTGGATGC
	TNF-α	GCCCATGTTGTAGCAAACCC	TATCTCTCAGCTCCACGCCA
	Bcl-2	GTACCTGAACCGGCATCTG	GGGGCCATATAGTTCCACAA
	Bax	TAGCAAACTGGTGCTCAAGG	TCTTGGATCCAGACAAGCAG
mice	Il-6	CTGGTGACAACCACGGCCTTCCC	ATGCTTAGGCATAACGCACTAG
		TA	GTT
	TNF-α	CACAAGATGCTGGGACAGTGA	TCCTTGATGGTGGTGCATGA
	β-actin	GACCTCTATGCCAACACAGTGC	GTACTCCTGCTTGCTGATCCAC

Table S6. Formulation of the high fat diet

Product #	D12492	
	gm (%)	kcal (%)
Protein	26.2	20
Carbohydrate	26.3	20
Fat	34.9	60
Total kcal/gm	100	
Ingredient	gm	kcal
Casein, 80 Mesh	200	800
L-cystine	3	12
Com starch	0	0
Maltodextrin 10	125	500
Sucrose	68.8	275.2
Fructose	0	0
Cellulose, BW200	50	0
Soybean Oil	25	225
Lard*	245	2205
Mineral Mix, S10026	10	0
DiCalcium Phosphate	13	0
Calcium Carbonate	5.5	0
Potassium Citrate, 1 H_2O	16.5	0
Vitamin Mix, V10001	10	40
Choline Bitartrate	2	0
FD＆C Blue Dye #1	0.05	0
Total	773.85	4057

*Typical analysis of cholesterol in lard = 0.95 mg/gram. Formulated by E. A. Ulman, Ph.D., Research Diets.

Cholesterol (mg)/4057 kcal = 232.8,

Cholesterol (mg)/kg = 300.8

Table S7. The qPCR raw data from the real-time RT-PCR assay in cells and animal samples.

**Experiment 1**	**Ct**			**a-ΔΔCt a**		**bF = $2^{-\Delta\Delta Ct}$**	
	**Control**	**10 μM A22**	**20 μM A22**	**10 μM A22**	**20 μM A22**	**10 μM A22**	**20 μM A22**
β-actin	12.46	12.29	12.41				
BCL-2	22.59	21.93	20.92	-0.66	-1.67	1.58	3.18
BAX	24.31	23.95	23.75	-0.36	-0.56	1.28	1.47

**Experiment 2**	**Ct**			**a-ΔΔCt a**		**bF = $2^{-\Delta\Delta Ct}$**	
	**Control**	**10 μM A22**	**20 μM A22**	**10 μM A22**	**20 μM A22**	**10 μM A22**	**20 μM A22**
β-actin	14.7	14.62	14.63				
C-KIT	31.18	31.06	31.16	-0.05	0.04	1.03	0.96
KRAS	24.13	24.03	24.03	-0.03	-0.04	1.02	1.03
VEGF	24.31	24.22	24.17	-0.01	-0.07	1.01	1.05
C-MYC	22.12	22.08	22.11	0.04	0.06	0.97	0.96

**Experiment 3**	**Ct**		**a-ΔΔCt a**	**bF = $2^{-\Delta\Delta Ct}$**
	β-actin	***BCL-2***		
Control	12.54	25.28	0	1
0.5 mM PA	13.53	27.84	1.57	0.33
0.5 mM PA+10 μM ***A22***	13.15	25.26	-0.63	1.54
0.5 mM PA+20 μM ***A22***	13.47	25.02	-1.19	2.28
0.5 mM PA+50 μM ***A22***	13.29	24.51	-1.52	2.87
	β-actin	***BAX***	**a-ΔΔCt a**	**bF = $2^{-\Delta\Delta Ct}$**
Control	12.54	21.89	0	
0.5 mM PA	13.53	22.78	-0.1	**1.0**
0.5 mM PA+10 μM ***A22***	13.15	22.79	0.29	**0.81**
0.5 mM PA+20 μM ***A22***	13.47	22.98	0.16	**0.89**
0.5 mM PA+50 μM ***A22***	13.29	22.5	-0.14	**0.90**

**Experiment 4**	**Ct**		**a-ΔΔCt a**	**bF = $2^{-\Delta\Delta Ct}$**
	β-actin	***BCL-2***		
CH	23.53	31.635	0	1
CH+40mg/kg ***A22***	24.145	32.33	0.08	0.946
HF	21.67	31.35	1.555	0.48
*HF+10mg/kg****A22***	23.185	30.26	-1.03	2.04
*HF+40mg/kg****A22***	22.135	28.535	-2.125	4.36
	β-actin	***BAX***	**a-ΔΔCt a**	**bF = $2^{-\Delta\Delta Ct}$**

CH	21.45	26.655	0	1
CH+40mg/kg ***A22***	21.335	26.795	0.255	0.8379
HF	24.055	28.015	-1.245	2.3701
*HF+10mg/kg**A22***	24.47	28.765	-0.91	1.8790
*HF+40mg/kg**A22***	23.965	28.645	-0.525	1.4389

**Experiment 5**	**Ct**	**[a]-ΔΔCt [a]**	**[b]F = $2^{-\Delta\Delta Ct}$**
Mock（IgG）	29.625	0	**1**
**20μM A22 HNRNP LL**	25.54	-4.085	16.971
**50μM A22 HNRNP LL**	23.293	-6.332	80.56

[a] **-ΔΔCt** = -[(Ct value of genes in treated sample - Ct value of genes in control) - (Ct value of GADPH in treated sample - Ct value of GADPH in control)]. [b] F: The relative expression fold.

Experiment 1: HepG2 cells were treated with different concentration of **A22**. The Ct values of *BCL-2* and Bax were obtained.

Experiment 2: HepG2 cells were treated with different concentration of **A22**. The Ct values of C-KIT, KRAS, VEGF, and C-MYC were obtained.

Experiment 3: The Ct values of *BCL-2* and Bax in 0.5 mM PA model with different concentration of **A22**.

Experiment 4: The Ct values of *BCL-2* and Bax in CH, HF, and **A22** administration group of mice liver.

Experiment 5: The Ct values of different chip samples in CHIP experiments.

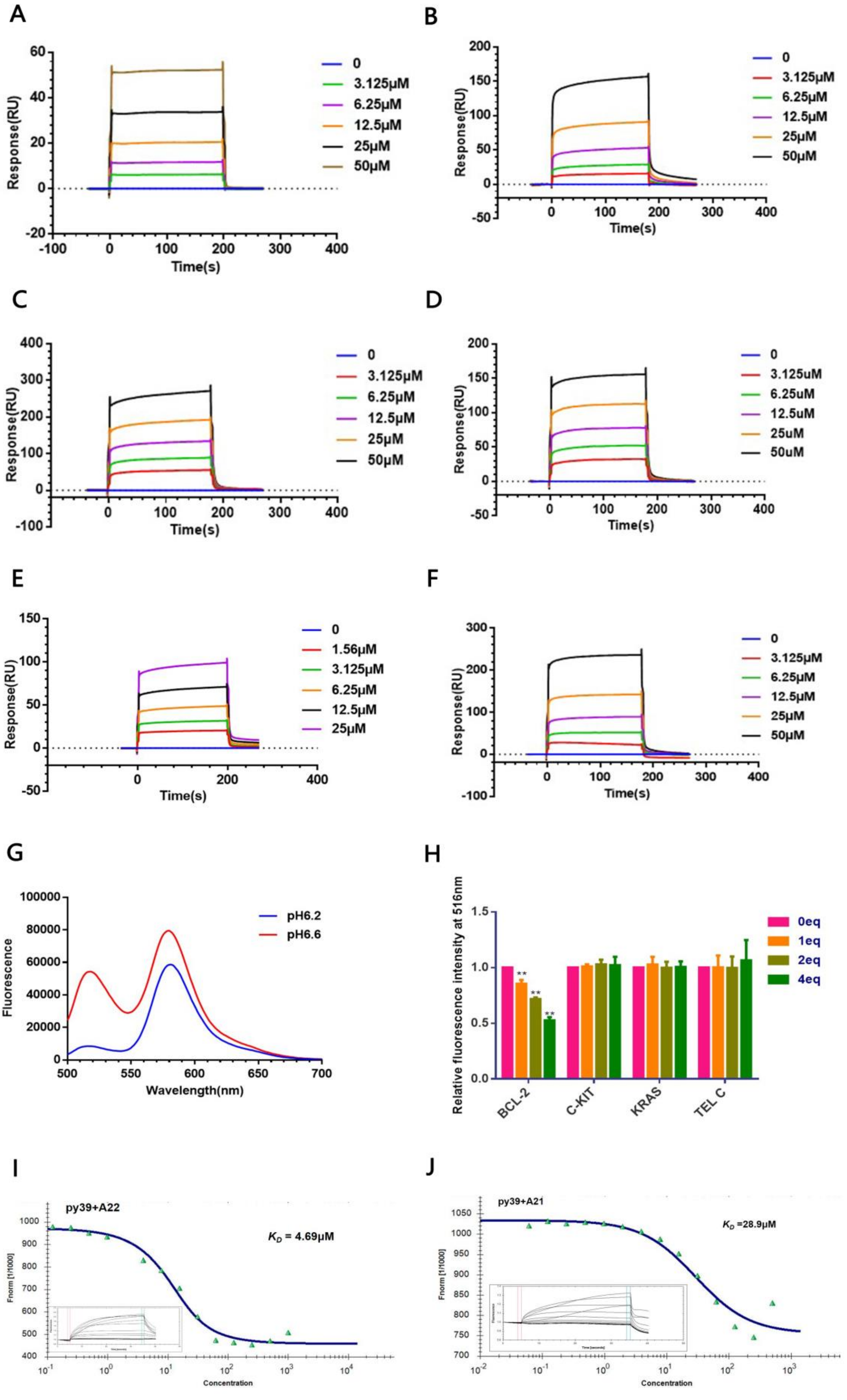
A
Response(RU)
Time(s)
0
3.125μM
6.25μM
12.5μM
25μM
50μM
B
Response(RU)
Time(s)
0
3.125μM
6.25μM
12.5μM
25μM
50μM
C
Response(RU)
Time(s)
0
3.125μM
6.25μM
12.5μM
25μM
50μM
D
Response(RU)
Time(s)
0
3.125uM
6.25uM
12.5uM
25uM
50uM
E
Response(RU)
Time(s)
0
1.56μM
3.125μM
6.25μM
12.5μM
25μM
F
Response(RU)
Time(s)
0
3.125μM
6.25μM
12.5μM
25μM
50μM
G
Fluorescence
Wavelength(nm)
pH6.2
pH6.6
H
Relative fluorescence intensity at 516nm
0eq
1eq
2eq
4eq
BCL-2
C-KIT
KRAS
TEL C
I
py39+A22
K_D = 4.69μM
Fnorm [1/1000]
Concentration
J
py39+A21
K_D =28.9μM
Fnorm [1/1000]
Concentration

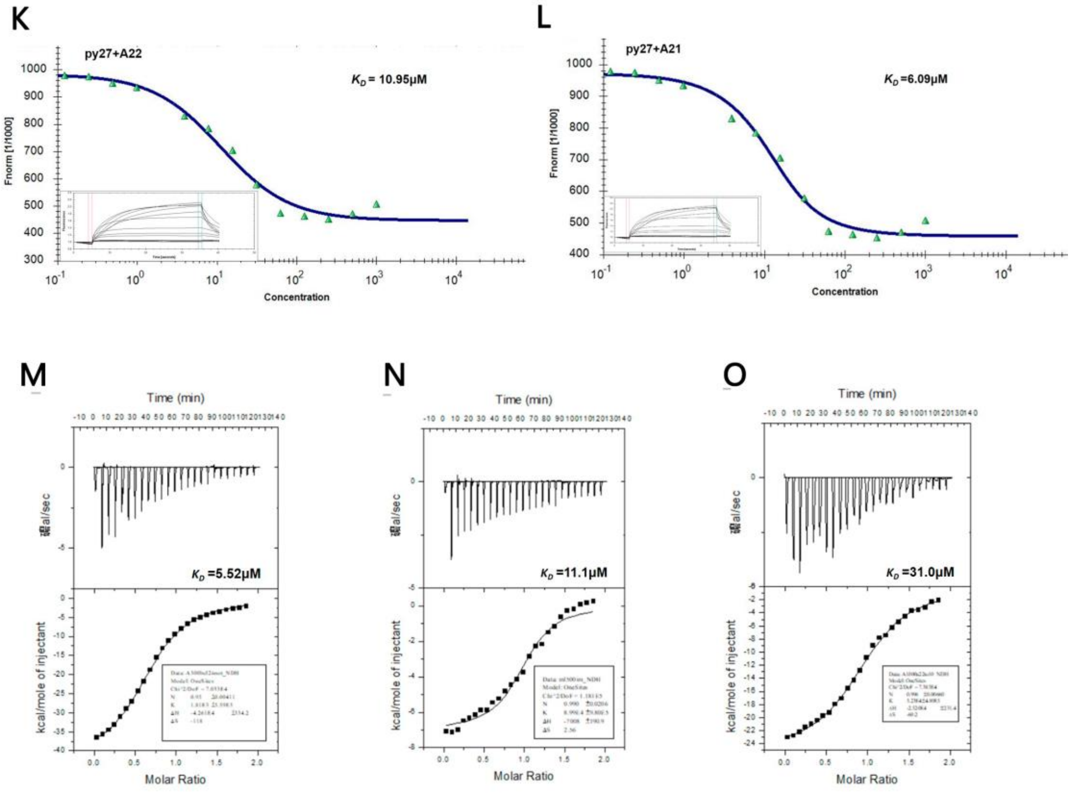

Figure S1. Biophysical experimental results for activity of acridine derivative in binding to *BCL-2* promoter i-motif and other DNA structures. (A) K_D value for binding of **A22** to *BCL-2* promoter G-quadruplex was estimated to be 48.9 μM measured by using SPR. Bio-pu39 was annealed in buffer of 20 mM Tris–HCl, 100 mM KCl, pH 7.4 in this experiment. (B)(C)(D)(E) SPR analyses for binding of **A22** to hairpin duplex DNA, *VEGF* promoter i-motif, *C-KIT* promoter i-motif, and *C-MYC* promoter i-motif, with their K_D values determined to be >50 μM, 36.4 μM, 34.5 μM, and 11.95 μM. Bio-hairpin was annealed in buffer of 20 mM Tris–HCl, 100 mM KCl, pH 7.4, and bio-VEGF, bio-C-KIT and bio-C-MYC were annealed in MES buffer at pH 5.5. (F) SPR experiment was performed to study interaction of **A21** to BCL-2 promoter i-motif, with its K_D value determined to be 40.3 μM. (G) pH-dependent FRET changes of *BCL-2* promoter i-motif dual labeled with FAM and TAMRA at the 5´-end and 3´-end, respectively. At low pH (pH 6.2), the I581/I516 ratio was 6.7. At high pH (pH 6.6), the I581/I516 ratio was 1.42, indicating that more i-motif structures were formed at pH 6.2. Therefore, pH

6.2 was selected for the follow-up FRET titration experiments of other dual labelled C-rich oligomers. (H) Dose-dependent spectra changes at 516 nm for various dual labeled C-rich oligomers (representing i-motifs from promoters of *BCL-2, C-KIT, KRAS, and Telomeric* DNA) could be observed with addition of increasing **A22** concentration, which were analyzed in 1XBPES buffer at pH 6.2. Dose-dependent decrease of absorbance at 516 nm for *BCL-2* indicated that **A22** could induce the formation of *BCL-2* promoter i-motif. All the experiments were repeated for three times. (I)(J)(K)(L) The K_D values were determined to be 4.69 μM, 28.9 μM, 10.95 μM, and 6.95 μM respectively through MST for interactions of py39+**A22**, py39+**A21**, py27+**A22**, and py27+**A21**. Fpy39 and Fpy27 were annealed in 1×BPES buffer at pH 5.5, and then pre-incubated with **A21** or **A22** at 25 °C for 1h. ITC experiments were also performed for interactions of py39 with **A22** (M), py27 with **A22** (N), and py39 with **A21** (O), and their K_D values were determined to be 5.52 μM, 11.1 μM, and 31.0 μM, respectively.

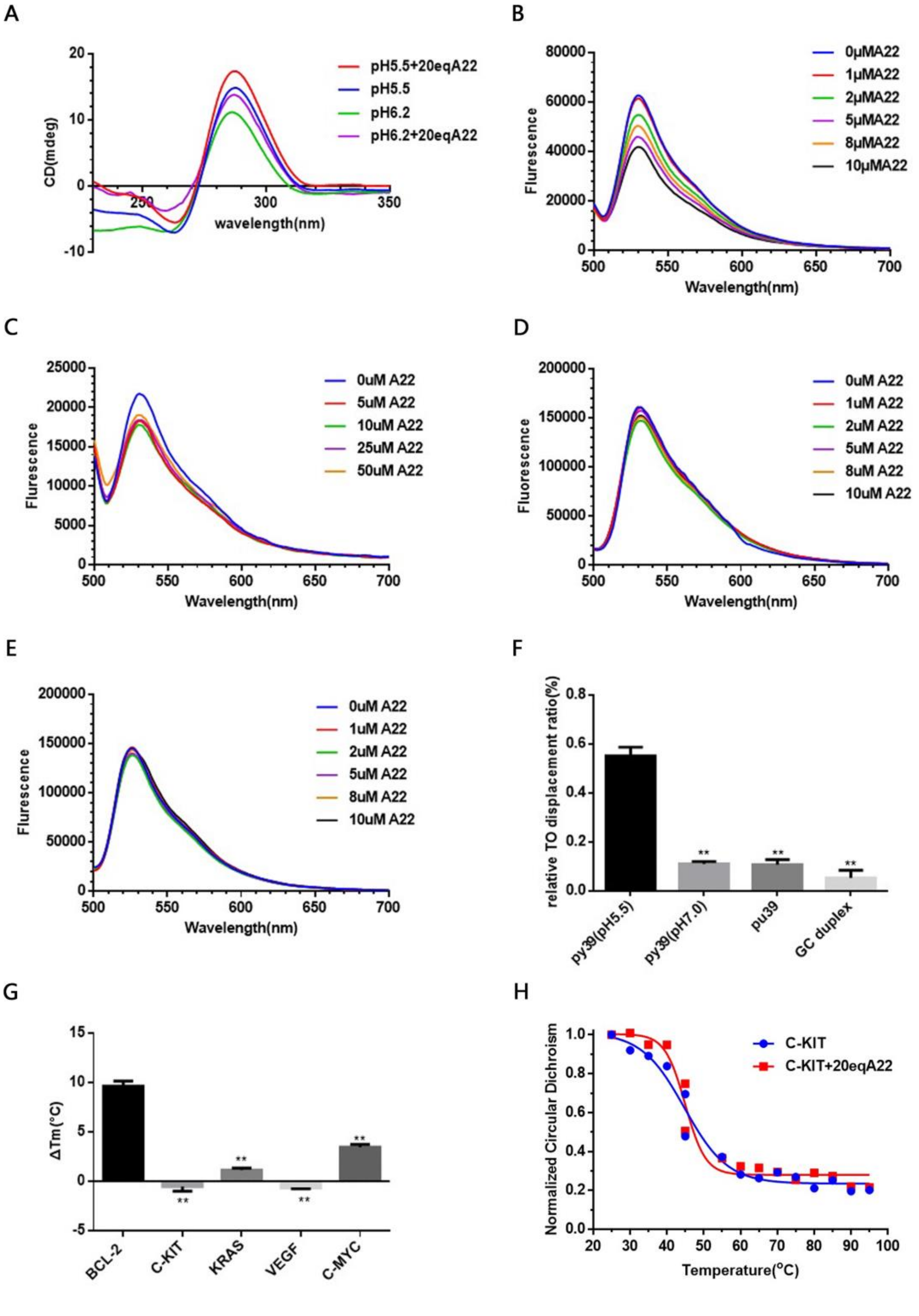
A
B
C
D
E
F
G
H
CD(mdeg)
wavelength(nm)
pH5.5+20eqA22
pH5.5
pH6.2
pH6.2+20eqA22
Flurescence
Wavelength(nm)
0μMA22
1μMA22
2μMA22
5μMA22
8μMA22
10μMA22
0uM A22
5uM A22
10uM A22
25uM A22
50uM A22
Fluorescence
1uM A22
2uM A22
5uM A22
8uM A22
10uM A22
relative TO displacement ratio(%)
py39(pH5.5)
py39(pH7.0)
pu39
GC duplex
ΔTm(°C)
BCL-2
C-KIT
KRAS
VEGF
C-MYC
Normalized Circular Dichroism
Temperature(°C)
C-KIT
C-KIT+20eqA22

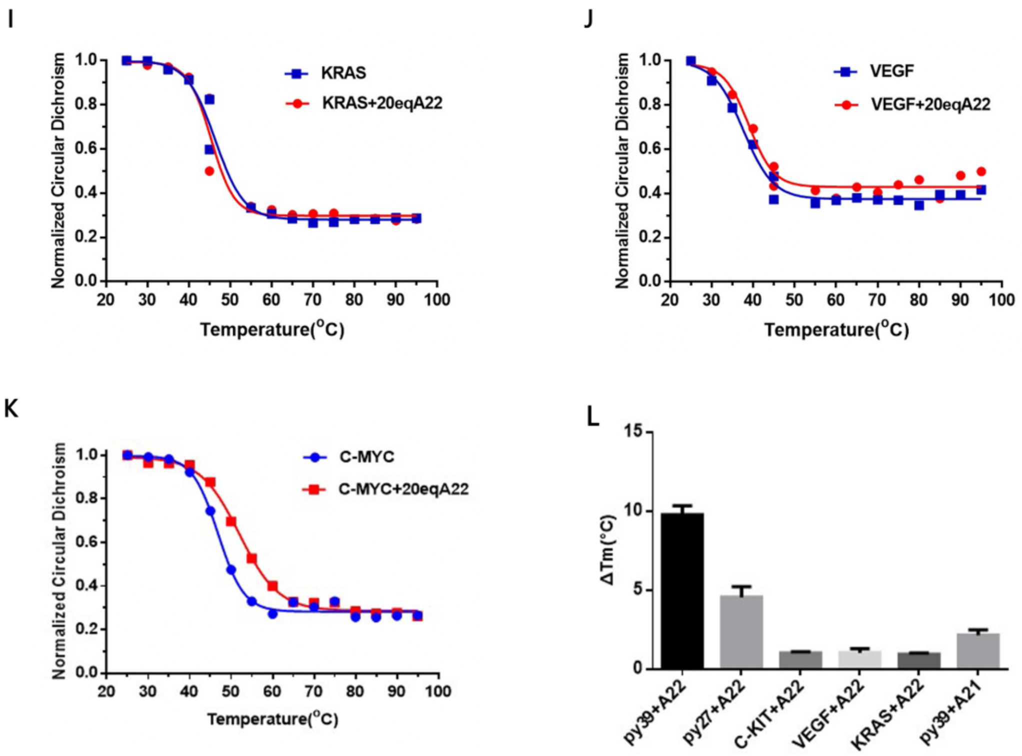

Figure S2. Effect of acridine derivative on oncogene promoter secondary structures analyzed by using various experiments. (A) CD spectra of BCL-2 promoter i-motif py39 at pH 6.2 and pH 5.5 with or without 20 equivalents of **A22**. The i-motif absorption bands were obviously increased in the CD spectra upon **A22** addition at both pH 6.2 and pH 5.5. (B-E) TO FIDS fluorescence spectra for binding of **A22** to py39 (annealed in 1XBPES buffer at pH 5.5), py39 (annealed in 1XBPES at pH 7.0), pu39 (BCL-2 promoter G-quadruplex, annealed in buffer of 20 mM Tris-HCl, 100 mM KCl, pH 7.4), and GC duplex (py39+pu39, annealed in buffer of 20 mM Tris-HCl, 20 mM NaCl, pH 7.4). (F) The above relative TO displacement ratio were statistically analyzed. (G) *ΔTm* values (°C) were determined by using FRET melting experiment for various types of i-motifs (F-py39-T, F-C-KIT-T, F-KRAS-T, F-VEGF-T, F-C-MYC-T) dual labeled with FAM and TAMRA at 5´- and 3´-end, annealed in 1XBPES buffer at pH 5.5. The concentration of DNA was 0.2 μM and the concentration of compound was 4 μM. The *ΔTm* values were determined to be 9.15 °C, -0.52 °C, 1.17 °C, -0.64 °C, and 3.50 °C for *BCL-2*, *C-KIT, KRAS, VEGF,* and *C-MYC* promoter i-motifs respectively. (H-K) CD melting experimental results for oncogene promoter i-motifs of *C-KIT, KRAS, VEGF*, *C-MYC* in the absence or

presence of 20 molar equivalents of **A22**. The oligonucleotides (*C-KIT, KRAS, VEGF, C-MYC*) were annealed in 1XBPES buffer at pH 5.5, with their *ΔTm* values (°C) determined to be 0.20 °C, 1.03 °C, 1.34 °C, and 4.26 °C, respectively. All these experiments were repeated for three times. (L) Graphical summary of *ΔTm* values (°C) determined by using CD melting experiments for various types of oligonucleotides with compounds.

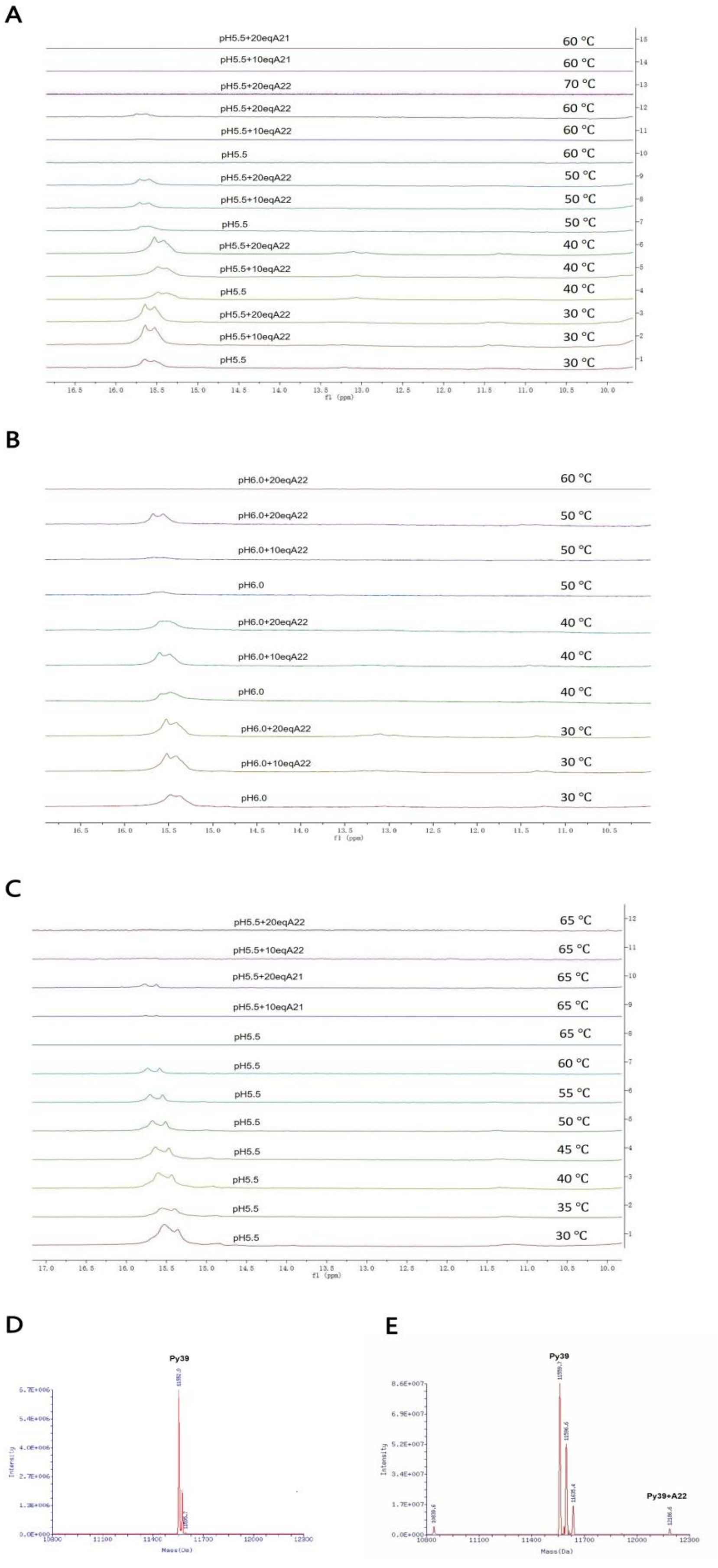
A
pH5.5+20eqA21 60 °C
pH5.5+10eqA21 60 °C
pH5.5+20eqA22 70 °C
pH5.5+20eqA22 60 °C
pH5.5+10eqA22 60 °C
pH5.5 60 °C
pH5.5+20eqA22 50 °C
pH5.5+10eqA22 50 °C
pH5.5 50 °C
pH5.5+20eqA22 40 °C
pH5.5+10eqA22 40 °C
pH5.5 40 °C
pH5.5+20eqA22 30 °C
pH5.5+10eqA22 30 °C
pH5.5 30 °C
f1 (ppm)
B
pH6.0+20eqA22 60 °C
pH6.0+20eqA22 50 °C
pH6.0+10eqA22 50 °C
pH6.0 50 °C
pH6.0+20eqA22 40 °C
pH6.0+10eqA22 40 °C
pH6.0 40 °C
pH6.0+20eqA22 30 °C
pH6.0+10eqA22 30 °C
pH6.0 30 °C
f1 (ppm)
C
pH5.5+20eqA22 65 °C
pH5.5+10eqA22 65 °C
pH5.5+20eqA21 65 °C
pH5.5+10eqA21 65 °C
pH5.5 65 °C
pH5.5 60 °C
pH5.5 55 °C
pH5.5 50 °C
pH5.5 45 °C
pH5.5 40 °C
pH5.5 35 °C
pH5.5 30 °C
f1 (ppm)
D
Py39
Intensity
Mass(Da)
E
Py39
Py39+A22
Intensity
Mass(Da)

Figure S3. NMR and ESI-MS spectra of DNA without or with the addition of acridine derivative. (A) The imino proton region of ^1H NMR spectra of oligomer py39 annealed in 1XBPES buffer at pH 5.5 upon addition of increasing concentration of **A22** at 30-70 °C**.** From bottom to top, spectra 1-3, 4-6, 7-9,10-12 were recorded for py39 with **A22** at various ratio (1:0, 1:10, 1:20) at temperatures of 30 °C, 40 °C, 50 °C, 60 °C respectively. Spectra 13 was recorded for py39: **A22** at ratio of 1:20 at 70 °C. Spectra 14 and 15 were recorded for py39 with 10 and 20 equivalents of **A21** respectively. (B) The imino proton region of ^1H NMR spectra for oligomer py39 annealed in 1XBPES buffer (pH 6.0) upon incubation with different equivalent of **A22** at 30-60 °C**.** From bottom to top, spectra 1-3, 4-6, 7-9 were recorded for py39 plus **A22** at various ratio (1:0,1:10,1:20) at temperatures of 30 °C, 40 °C, 50 °C, respectively. Spectrum 10 was recorded for py39 plus **A22** at ratio of 1:20 at 60 °C. (C) The imino proton region of ^1H NMR spectra of oligomer py27 annealed in 1XBPES buffer at pH 5.5 at 30-65 °C**.** The characteristic of C-MYC promoter i-motif disappeared at 65 °C. Upon addition of 20 equivalents of **A21**, a peak appeared at 15-16 ppm in imino proton region, suggesting that **A21** could induce the formation of C-MYC promoter i-motif and stabilize the secondary structure at 65 °C. In contrast, no peak was observed upon addition of 10 and 20 equivalents of **A22** at 65 °C**.** (D) ESI-MS spectrum for oligomer py39 only in 1XBPES buffer at pH 7.0. (E) ESI-MS spectra for oligomer py39 with 20 equivalents of **A22** in 1XBPES buffer at pH 7.0. All these experiments were repeated for three times.

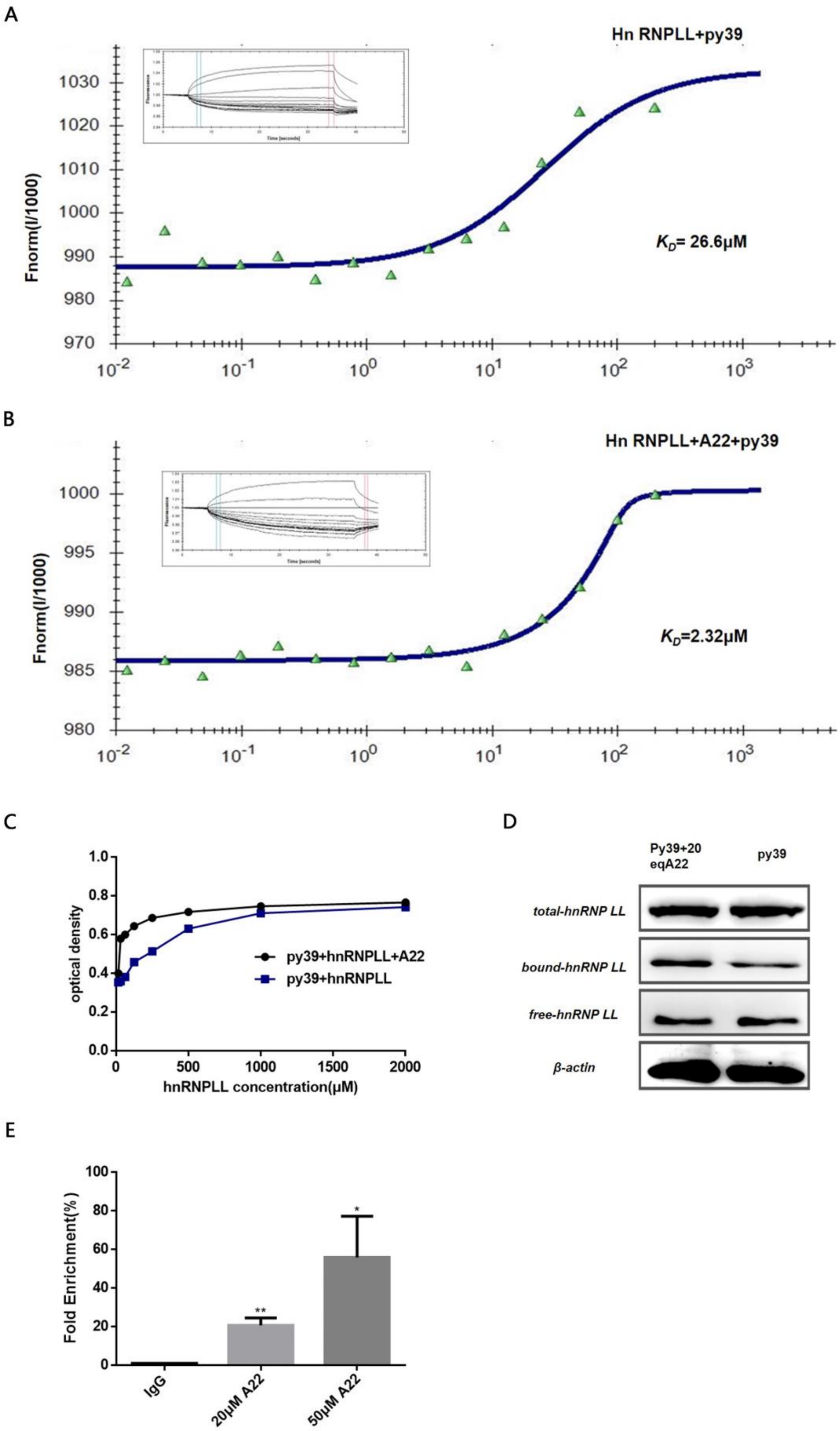
A
Hn RNPLL+py39
Fnorm(I/1000)
K_D= 26.6μM
B
Hn RNPLL+A22+py39
Fnorm(I/1000)
K_D=2.32μM
C
optical density
py39+hnRNPLL+A22
py39+hnRNPLL
hnRNPLL concentration(μM)
D
Py39+20 eqA22
py39
total-hnRNP LL
bound-hnRNP LL
free-hnRNP LL
β-actin
E
Fold Enrichment(%)
IgG
20μM A22
50μM A22

Figure S4. Effect of **A22** on the interaction of *BCL-2* promoter i-motif with its binding protein hnRNP LL. (A) MST result for the binding of fluorescently labeled *BCL-2* promoter i-motif to hnRNP LL. Fpy39 was annealed in 1XBPES buffer at pH 5.5. (B) MST result for the binding of fluorescently labeled *BCL-2* promoter i-motif to hnRNP LL, with the i-motif pre-incubated with **A22** at 25 °C for 1h. Fpy39 was annealed in 1XBPES buffer at pH 5.5. (C) Elisa result for the binding of *BCL-2* promoter i-motif to hnRNP LL in the absence or presence of **A22**. Bio-py39 was annealed in 1XBPES buffer at pH 5.5. (D) DNA pull down experiment was carried out by using magnetic beads. After addition of 20 eq **A22**, the amount of hnRNP LL bound to biotin-labeled *BCL-2* promoter i-motif was significantly higher than the control group without **A22**. Bio-py39 was annealed in 1XBPES buffer at pH 5.5. (E) Recruitment of hnRNP LL to *BCL-2* promoter P1 i-motif on chromosomes in **A22** treated HepG2 cells. The Fold Enrichment Method was used to analyze ChIP-qPCR data relative to input as this includes normalization for both background levels and input chromatin going into the ChIP. **A22** could significantly increase hnRNP LL proteins interacting with *BCL-2* promoter i-motif in a dose-dependent manner. All the experiments were repeated for three times. The data are expressed as the mean ± SEM: (*) $P < 0.05$, (**) $P < 0.01$, significantly different from the control.

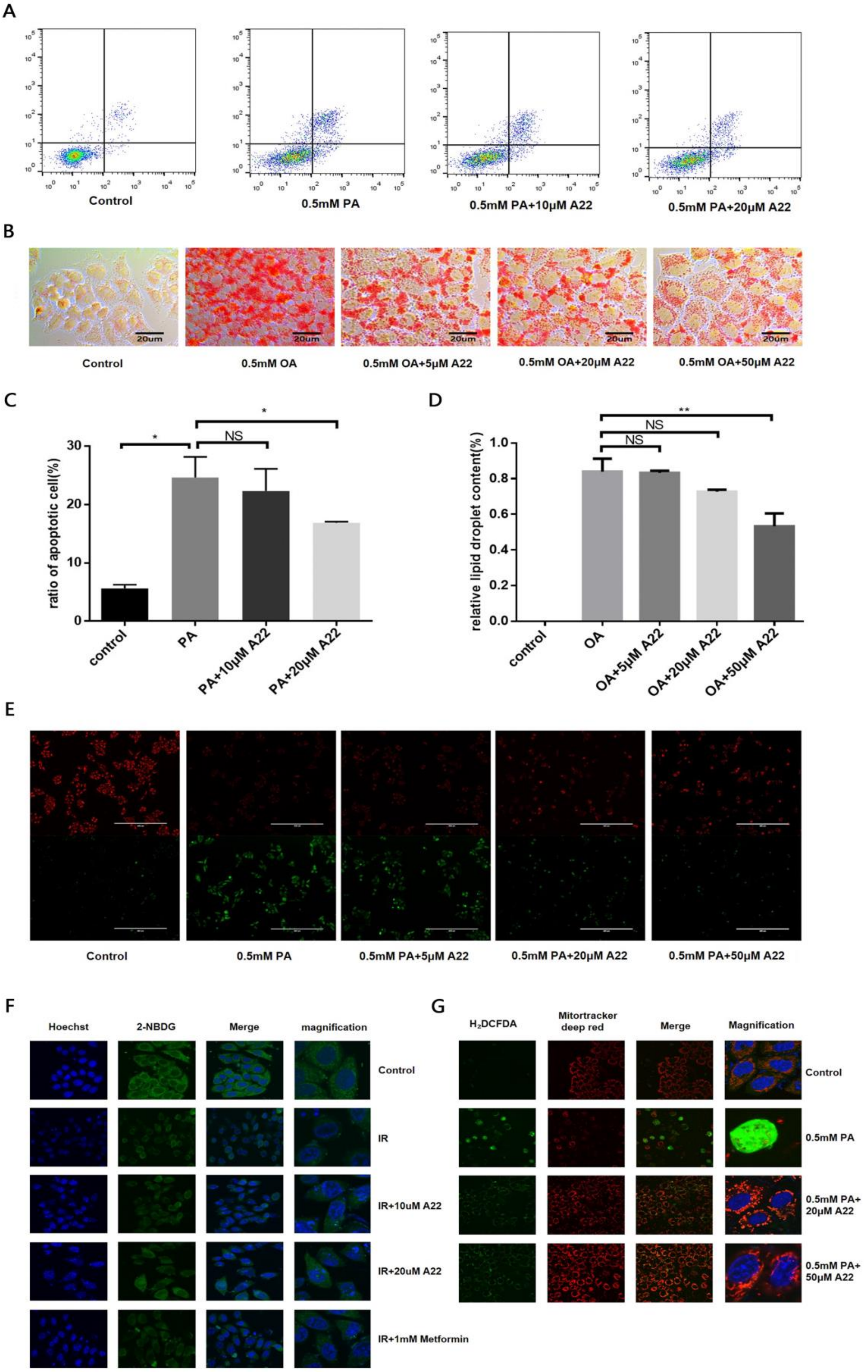

Figure S5. **A22** could reduce hepatocyte apoptosis, lipid deposition, improve glucose

uptake and release endoplasmic reticulum stress *in vitro*. (A) FITC Annexin V/PI experiments were carried out with flow cytometry. HepG2 cells were treated with 0.5 mM palmitic acid (PA) for 24hr. On the basis of PA induced model, 10 and 20 μM **A22** were added to the model. (B) Representative images of **A22** treatment in 0.5 mM oleic acid (OA) induced droplet formation. (C) Apoptosis ratio for FITC/PI experiment (A) in the absence or presence of **A22**. (D) Relative lipid droplet content for experiment (B) in the absence or presence of **A22**. The content of triglycerides in cells was analyzed by using a TG detection kit. The content of intracellular lipid deposition was assayed by using a TG detection kit. Cells were cultured and lysed with RIPA after treatment with 0.5 mM OA for 24 hours. The supernatant was centrifuged, the reagent was added in turn, and the OD values of all samples were measured with the microplate reader. Meanwhile, the protein concentration of each sample was also measured. TG content (mmol/gprot) = (ODsample - ODblank) / (ODcalibration - ODblank) *2.26 mM + protein concentration (gprot/L). (E) Representative images of 0.5 mM PA induced apoptosis models treated with increasing concentration of **A22** followed with JC-1 staining, indicating mitochondrial membrane potential in early apoptosis. (F) **A22** relieved the impaired glucose uptake of hepatic cells in the insulin resistance model by 2-NBDG (2-(N-(7-nitrobenz-2-oxa-1,3-diazol-4-yl)amino)-2-deoxyglucose). 2-NBDG is a fluorescent glucose analog that has been used to monitor glucose uptake in live cells, as an indicator of Hepatocyte viability and function. (G) Representative images of 0.5 mM PA induced apoptosis models treated with increasing concentration of **A22** followed with H_2DCFDA staining for detection of reactive oxygen species (ROS) and Mitortracker deep red probe of mitochondria in live cells. All the experiments were repeated for three times. The data are expressed as the mean ± SEM: (*) $P < 0.05$, (**) $P < 0.01$, significantly different from the control.

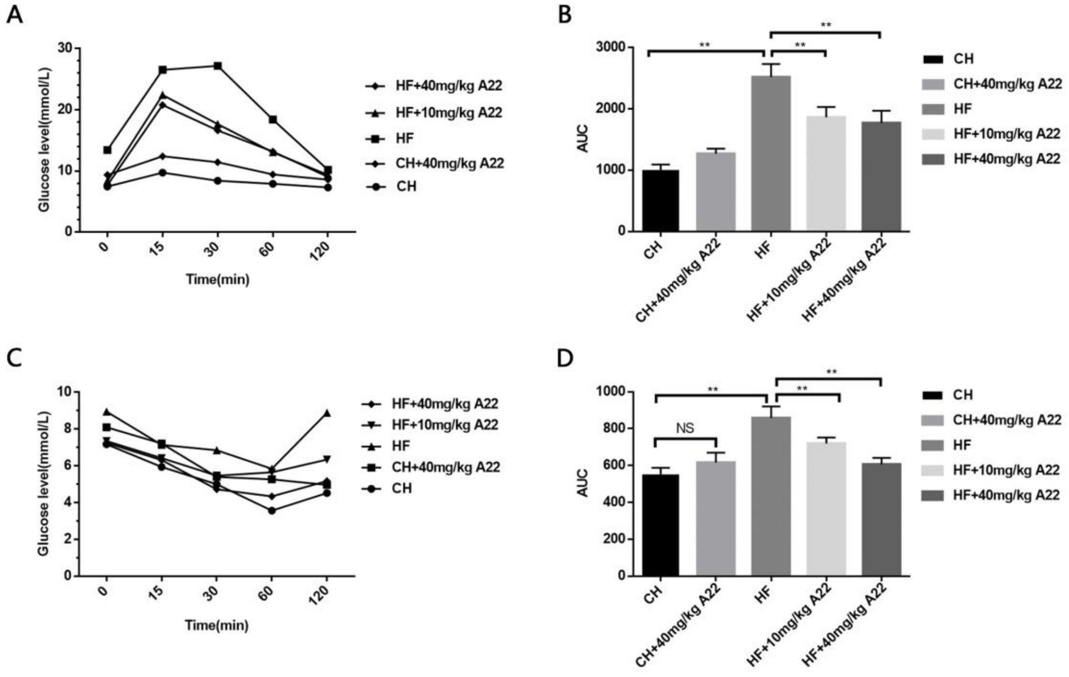

Figure S6. Glucose Tolerance Tests of NAFLD/NASH mice. Glucose Tolerance Tests were carried out after 4 weeks **A22** treatment as described. Compared with chow diet (CH) group, fasting blood glucose in high diet (HF) group was significantly higher. (A) After administration of glucose (20% solution) through intraperitoneal injection in overnight-fasted mice, glucose level was tested by cutting 1–2 mm of tissue from the tail tip. Glucose levels (mM) were recorded over a period of 2 hours. (B) $AUC_{glucose}$ data are presented. AUC means Area Under Curve. (C) After administration of insulin (0.5 U/kg) by intraperitoneal injection in overnight-fasted mice, glucose level was recorded for the next 2 hours. (D) $AUC_{insulin}$ data are presented. Data are statistically analyzed as means ± SEM, and each spot indicated one group (N = 8 mice/group). *p<0.05, vs. HF control mice; **p<0.01, vs. HF control group.

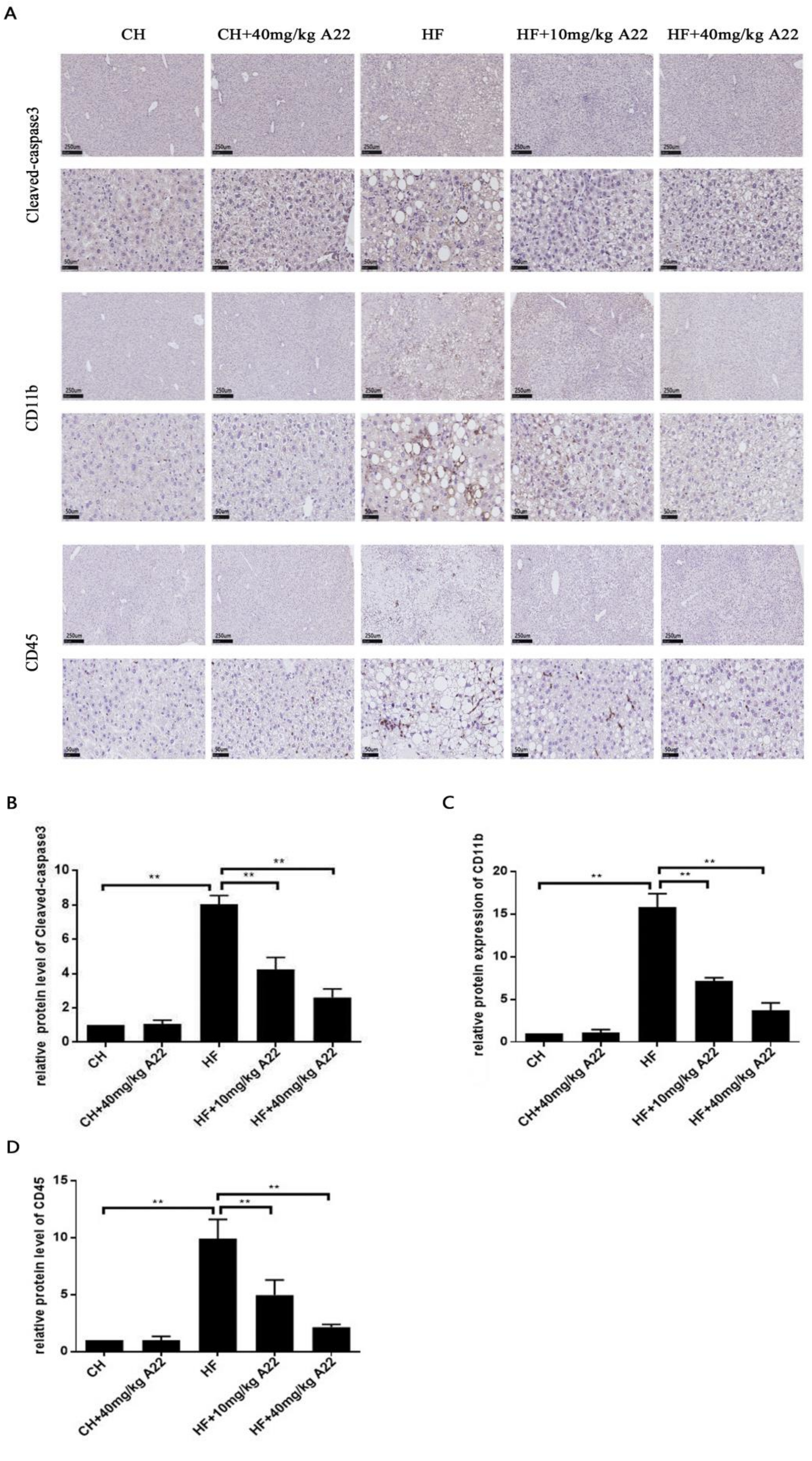
A
CH
CH+40mg/kg A22
HF
HF+10mg/kg A22
HF+40mg/kg A22
Cleaved-caspase3
CD11b
CD45
B
relative protein level of Cleaved-caspase3
0
2
4
6
8
10
**
**
**
CH
CH+40mg/kg A22
HF
HF+10mg/kg A22
HF+40mg/kg A22
C
relative protein expression of CD11b
0
5
10
15
20
**
**
**
CH
CH+40mg/kg A22
HF
HF+10mg/kg A22
HF+40mg/kg A22
D
relative protein level of CD45
0
5
10
15
**
**
**
CH
CH+40mg/kg A22
HF
HF+10mg/kg A22
HF+40mg/kg A22

Figure S7. **A22** inhibited lipid-induced liver apoptosis and inflammation in immunohistochemistry stain. (A) Representative images of cleaved-caspase3, CD11b, and CD45 of immunohistochemistry (IHC). Data are statistically analyzed as shown in (B) (C) (D) as means ± SEM, and each column indicated one group (N = 8 mice/group). *p<0.05, vs. HF control mice; **p<0.01, vs. HF control group.

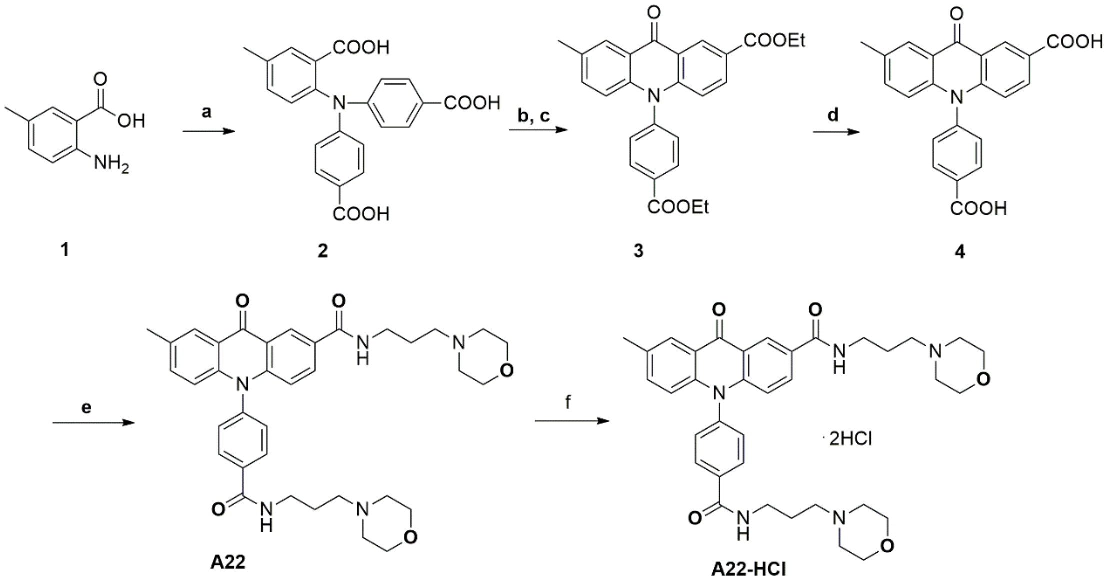

Scheme I Synthetic route for acridone **A22** and **A22-HCl**. Reagents and conditions: (a) 4-iodobenzoic acid, Cu, CuI, K_2CO_3, DMF, 120°C, 36 h; (b) conc. H_2SO_4, 140 °C, 3h; (c) EtOH, 80 °C, 2 h (yield, 17% for three steps); (d) MeOH, 10% NaOH, 60 °C, 1 h (yield 91%); (e) TCM, T_3P, 3-morpholinopropan-1-amine, r.t., 2 h (yield, 88%); (f) MeOH, methanolic hydrochloric acid solution, r.t., 3 h (yield, 98%).

MATERIAL AND METHODS

General materials

Chemically synthesized DNA oligomers of HPLC purified grade were purchased from Sangon Biotec. All oligonucleotide concentrations were determined by measuring absorbance at 260 nm using a Nano Drop1000 spectrophotometer (Thermo Scientific). For obtaining i-motif structures, C-rich oligonucleotides were annealed in 1XBPES buffer (30 mM KH_2PO_4, 30 mM K_2HPO_4, 1 mM EDTA, 100 mM KCl) with different pH at 95 ^oC for 5 min, and then cooled to room temperature. For obtaining G-quadruplex, oligonucleotides were annealed in 20 mM Tris-HCl buffer containing 100 mM KCl (pH 7.0) by heating at 95 ^oC for 5 min followed with gradual cooling to room temperature. Formation of secondary non-B DNA structures were determined by using circular dichroism (CD) spectrophotometer.

Electrospray mass spectrometry (ESI-MS) experiment

Py39 oligonucleotides at concentration of 5 μM were annealed in 1XBPES buffer with or without 20 μM **A22** at 95 °C for 5 min, followed with cooling to room temperature. Mass spectrometry signals were collected by using ESI-MS (Thermo, LCQ DECA Plus XP) and analyzed by using pro-mass.

Fluorescence resonance energy transfer (FRET) melting experiment

FRET melting assay was performed on a real-time PCR apparatus (Roche Light Cycler 2) as follows. The oligonucleotides with fluorescent 5´-FAM and 3´-TAMRA labeling at concentration of 400 nM in different buffers were annealed by heating at 95 °C for 5 min followed by cooling to room temperature. DNA Fluorescence melting curves of 0.2 μM labeled oligonucleotide were recorded at an interval of 1 °C over the range of 37−95 °C. Measurements were carried out in Light Cycler capillaries with excitation at 470 nm and detection at 530 nm for triplicate. Samples were incubated with or without **A22** for 1h at 25 °C before measurement. Data were analyzed by using Origin 8 (Origin Lab Corp) software.

Microscale thermophoresis experiment

5′-FAM-labelled py39 (1 μM) was annealed in 1XBPES buffer by heating at 95 °C for 5 min in the absence or presence of 20 equivalent **A22**. Protein hnRNP LL initial

concentration was 2 nM, and half-diluted 15 times in PBS buffer (pH 7.4, 0.05% Tween-20). After incubation for 60 min, the samples were loaded in microscale thermophoresis (MST)-grade glass capillaries. The intensity of the LED power was 20% and the MST laser was set at 40%. The analyses were performed using a Monolith NT.115, and the fitting curve was obtained by using NT Analysis 1.5.41 via Hill fitting. The K_D value represents the numeric equivalent of the concentration of hnRNP LL when the response is half of the plateau response (Rmax) of the fitting curve. In the MST experiments of DNA with small molecule, 5′-FAM-labelled DNA (1 μM) was annealed in 1XBPES (pH 5.5) buffer by heating at 95 °C for 5 min. The concentration of compound was 50 μM, and half-diluted 15 times in 1XBPES buffer. After incubation of DNA with compound for 60 min, the sample was loaded in microscale thermophoresis (MST)-grade glass capillaries. The intensity of the LED power was 20% and the MST laser was set at 40%. The analysis method was the same as above.

Isothermal titration calorimetry (ITC)

The interactions between compound and DNA were measured using isothermal titration calorimetry (ITC) with a VP isothermal titration calorimeter from Microcal, Inc. (Northampton, USA). The DNA was annealed in 1XBPES (pH 5.5) buffer by heating at 95 °C for 5 min and then cooled to room temperature. Calorimetric titrations of **A21** or **A22** (0.5 mM in the syringe) to DNA (0.05 mM in the cuvette) were carried out at 25 °C in 1XBPES (pH 5.5) buffer. The compound was titrated into DNA in 5 μL injections with a spacing of 300 s between injections. Calorimetric data were analyzed by integrating heat effects normalized to the amount of injected compound and curve-fitting based on a 1:1 binding model using the Origin software package (Microcal). The binding constant was derived from data using standard procedures.

Purification of recombinant protein hnRNP LL

hnRNP LL cDNA was synthesized by Sangon Biotech company, and cloned into DH5α protein expression vector (Tiangen). This constructed plasmid was transformed into BL21(DE3) after sequencing analysis. 0.1 mM IPTG (isopropyl β-D-1-thiogalactopyranoside) was used to induce the expression of hnRNP LL protein

overnight at room temperature. Harvested cells were resuspended in a lysis buffer (20 mM Na_3PO_4, pH 7.4, 500 mM NaCl, 1% Triton X-100, 1 mg/mL lysozyme, and 1× protease inhibitor cocktail [Sigma, #8465]), vortexed for 90s, and sonicated for 30min (cycle of 3s on and 7s off) on ice. Cell debris was removed through centrifugation at 12,000 rpm for 50min at 4 °C. The supernatant was collected, and applied to Ni affinity column. After washing with binding buffer, protein hnRNP LL was eluted with eluting buffer. The concentration of hnRNP LL was determined by using ultramicro-UV and SDS-PAGE. Purified hnRNP LL was stored in buffer of 20 mM HEPES-NaOH (pH 7.4), 100 mM KCl, 10% glycerol, 2 mM DTT, and 0.1% NP-40 at -80 °C for experimental use.

DNA Pull down experiment

Biotin labeled py39 was annealed at 95 °C for 5 minutes with or without 20 equivalents of **A22** in 1XBPES. The annealed DNA was incubated with cell lysate for 1.5hr at 37 °C. Then the mixture was incubated with magnetic bead (Thermo Fisher Dynabeads™ Streptavidin Trial Kit, 65801D) for 1hr at room temperature. The bead was washed with PBS (pH 7.4) buffer on a magnetic stand to remove non-adhering and low-specificity DNA-binding protein. Finally, the amount of *BCL-2* promoter DNA specific binding protein was analyzed by using Western Blot.

RNA extraction and qRT-PCR

Total RNA from cells and mouse livers were isolated by using RNAiso Plus (Takara, 9109, Japan). After homogenizing the sample, chloroform was added, and the homogenate was allowed to separate into a clear upper aqueous layer (containing RNA), an interface, and a red lower organic layer (containing the DNA and proteins). RNA was precipitated from the aqueous layer with isopropanol. DNA was precipitated from the aqueous/organic interface with ethanol. cDNA was synthesized with a One-Step RT-PCR Kit (Takara, Cat#639503, Japan).

Quantitative real-time polymerase chain reaction (PCR) was carried out using 2×RealStar SYBR Mixture (GenStar, Cat# A301-10, Guangzhou, China). The results were analyzed on a LightCycler480 II real-time PCR system (ROCHE, USA) using the

2-ΔΔCt method. Primers were synthesized by Sangon Biotec (Guangzhou, China) and primers sequences were listed in Table S5. Actin was used as a loading control and relative mRNA levels were normalized to Actin.

Western blotting

Cells or liver simples were lysed in cold RIPA extraction buffer (Beyotime, Cat# P0013C, Chengdu, China) with an addition of protease inhibitors (Roche, Cat# 4693006001, Guangzhou, China). The extracted proteins were separated by SDS-PAGE and transferred to a polyvinylidene difluoride membrane (Millipore, Guangzhou). After blocking with TBS/T (0.1%) containing 5% bovine serum albumin (BSA) for 25-30 min at the room temperature, the membrane was incubated with different primary antibodies of BCL-2 (affinity biosciences OH, Cat# AF6139, USA), BAX (affinity biosciences OH, Cat# AF0120, USA), VEGF (affinity biosciences OH, Cat# AF5131, USA), C-KIT (affinity biosciences OH, Cat# AF6153, USA), C-MYC (affinity biosciences OH, Cat# AF0358, USA), Caspase9 (affinity biosciences OH, Cat# AF6348, USA), cleaved-Caspase9 (affinity biosciences OH, Cat# AF5240, USA), Caspase3 (affinity biosciences OH, Cat# AF6311, USA), cleaved-Caspase3 (affinity biosciences OH, Cat# AF7022, USA), PARP (affinity biosciences OH, Cat# AF6139, USA),cleaved-PARP (affinity biosciences OH, Cat# AF6139, USA), IRE 1α (Cell Signaling Technology, Cat# 3294T, USA), PERK (Cell Signaling Technology, Cat# 3192S, USA), eIF-2α (Cell Signaling Technology, Cat# 5324S, USA), P-eIF-2α (Cell Signaling Technology, Cat# 3298T, USA), CHOP (Cell Signaling Technology, Cat# 2895T, USA), IL-6 (Cell Signaling Technology Cat# 12153, USA), TNF-α (Cell Signaling Technology Cat# 6945, USA), NF-κB (Cell Signaling Technology Cat# 6956, USA), α-SMA (Cell Signaling Technology, Cat# 19245T, USA), Collagen I (Cell Signaling Technology, Cat# 91144S, USA) by 1:1,000 dilution in 5% bovine serum albumin at 4 °C overnight. The membrane was washed with TBS/T for 4 x 10 min to remove unbound antibody, and then incubated with HRP-conjugated secondary antibodies (Cell Signaling Technology, Cat# 7076 from mouse and Cat# 7074 from rabbit, China). Protein bands were visualized with an ECL kit (Millipore, Cat# 64-201BP, China). Densitometry analysis was performed using Quantity One

Software (Bio-Rad Laboratories, CA, USA) relative to the loading control.

Chromatin immunoprecipitation (ChIP)

Chromatin immunoprecipitation (ChIP) experiments were performed by using a Pierce Magnetic ChIP Kit (Thermo Fisher) according to manufacturer's protocol. Briefly, HepG2 cells with or without **A22** were fixed with 1% formaldehyde for 10 min and then scraped and transferred to a microcentrifuge tube. After centrifugation, 10% of the lysate was removed as an input sample, and 10 μg of antibody against hnRNP LL (Affinity) was used for ChIP. Normal rabbit IgG (Thermo Fisher) was used as the negative control. CHIP mixture was incubated at 4 °C overnight. The immune complexes were collected with protein A magnetic beads. After repeated washing, purified DNA was extracted from immunoprecipitated chromatin and amplified by using qRT-PCR.

FITC Annexin V/PI apoptosis detection

FITC Annexin V/PI apoptosis detection was performed with the FITC Annexin V/PI Apoptosis Detection Kit (Muti sciences, 70-AP101-100). Cells in 6 well plate were treated with different concentration of **A22** in PA model, and then digested and resuspended in 500 μL binding buffer. FITC Annexin V (5 μL) was incubated with samples for 5 mins, and then 5 μL PI was added for incubation at room temperature for 10 min. The fluorescence was analyzed by using flow cytometry (Millipore, guava easy Cyte), and data were analyzed by using Flow jo V10.

Hoechst 33342/Mitortracker deep red/H2DCFDA/JC-1 staining and confocal imaging

HepG2 cells were plated on 96 well plates. After 24h treatment with **A22** in 0.5 mM PA induced model or insulin resistance cell model, the cells were washed for three times with cold PBS. Then cells were incubated with Hoechst 33342 solution (Sigma, 5 g/mL) and H2DCFDA probe (10 μM, sigma), as well as Mitortracker deep red (100 nM, sigma) for 10 min at 37 °C in the dark. For insulin resistance model, 2-NBDG (10 μM, sigma), a fluorescent indicator for direct glucose uptake, and Hoechst 33342 were incubated with HepG2 cells. All the samples were photographed by using FV3000 (Olympus). HepG2 cells were plated on 6 well plates. After 24h

treatment with **A22** in 0.5 mM PA induced model, cells were incubated with JC-1 (5 μg/mL, sigma) for 30 min at 37 °C in the dark. Fluorescence detection was carried out by using EVOS FL Auto.

Oil red O staining and TG detection

After treated with 0.5 mM OA for 24 hours with or without different concentration of **A22**, cells were fixed with 4% paraformaldehyde at room temperature for 1 hr and then stained with fresh 0.5% Oil Red O solution for 30 min in dark place. After the staining, cells were rinsed with 60% isopropanol, washed with distilled water and analyzed under EVOS FL Auto. Cellular TG were extracted and determined with instruction of commercial Peridochrom TG GPO-PAP kit (Jiancheng Bio, Cat# A110-2, Nanjing, China). Data were collected and analyzed by image J.

Glucose and Insulin Tolerance Tests in mice

The *in vivo* metabolic tests are greatly valuable to evaluate whether metabolic syndrome in mouse models are accompanied by abnormal carbohydrate metabolism such as glucose intolerance and insulin resistance. Glucose tolerance test (GTT) was performed to measure the clearance ability of body through an intraperitoneal injected glucose load in overnight-fasted mice. Subsequently plasmatic glucose levels were measured every 30 minutes during the following 2h. The final dose of intraperitoneal injection was 2g of glucose/kg of BW. The insulin tolerance test (ITT) assesses insulin sensitivity by monitoring endogenous blood glucose disappearance timely in response to exogenous injection of human insulin, which was performed in mice fasted for 4-6h with a dose of 0.5 U/kg insulin of BW. Glucose levels were measured as above. Homeostasis model assessment-insulin resistance index (HOMA – IR) = (FPG × Fins)/22. 5.

Transferase-mediated dUTP-biotin nick end labeling (TUNEL) assay

Mice liver samples were fixed in 10% paraformaldehyde and embedded in paraffin. All the samples were stripped of protein by incubating with 20 mg/mL proteinase K (Servicebio, G1205, Guangzhou, China) for 15 min at room temperature after de-paraffinisation. TUNEL staining was performed with an in-situ apoptosis detection kit (Roche, Cat#11684817910, Guangzhou, China) according to the

manufacture's protocols. The nuclei were stained with Hematoxylin dye (Servicebio, Cat#G1004), and TUNEL-positive cells were visualized by using a light microscope (Olympus, Germany) and photographed at X40 magnification. TUNEL labeled cells in each slide were calculated with Image J software (Macbiophotonics, McMaster University, RRID: SCR_003070) by Servicebio Company (Beijing, China).

Histological Scoring for mice liver

The histological feature of animal livers was scored validating a scoring system which addresses the full spectrum of lesions of NAFLD and proposed a NAFLD activity score (NAS) for use in clinical trials. It is recommended by the Pathology Committee of the NASH Clinical Research Network. 14 histological features were compromised in the scoring system: 4 of which were evaluated semi-quantitatively: steatosis (0-3), lobular inflammation (0-2), hepatocellular ballooning (0-2), and fibrosis (0-4). Other nine features were recorded as present or absent. The score ≥5 means a diagnosis of NASH, and tissue samples with scores of less than 3 were diagnosed as “not NASH.”
